# Power laws and critical fragmentation in global forests

**DOI:** 10.1038/s41598-018-36120-w

**Published:** 2018-12-10

**Authors:** Leonardo A. Saravia, Santiago R. Doyle, Ben Bond-Lamberty

**Affiliations:** 1 Instituto de Ciencias, Universidad Nacional de General Sarmiento, J.M. Gutierrez 1159 (1613), Los Polvorines, Buenos Aires, Argentina; 2 Pacific Northwest National Laboratory, Joint Global Change Research Institute at the University of Maryland–College Park, 5825 University Research Court #3500, College Park, MD 20740 USA

## Abstract

The replacement of forest areas with human-dominated landscapes usually leads to fragmentation, altering the structure and function of the forest. Here we studied the dynamics of forest patch sizes at a global level, examining signals of a critical transition from an unfragmented to a fragmented state, using the MODIS vegetation continuous field. We defined wide regions of connected forest across continents and big islands, and combined five criteria, including the distribution of patch sizes and the fluctuations of the largest patch over the last sixteen years, to evaluate the closeness of each region to a fragmentation threshold. Regions with the highest deforestation rates–South America, Southeast Asia, Africa–all met these criteria and may thus be near a critical fragmentation threshold. This implies that if current forest loss rates are maintained, wide continental areas could suddenly fragment, triggering extensive species loss and degradation of ecosystems services.

## Introduction

Forests are among the most important biomes on earth, providing habitat for a large proportion of species and contributing extensively to global biodiversity^1^. In the previous century, human activities have influenced global bio-geochemical cycles^2,3^, with one of the most dramatic changes being the replacement of 40% of Earth’s formerly biodiverse land areas with landscapes that contain only a few species of crop plants, domestic animals and humans^4^. These local changes have accumulated over time and now constitute a global forcing^5^. Another global scale forcing that is tied to habitat destruction is fragmentation, which is defined as the division of a continuous habitat into separated portions that are smaller and more isolated. Fragmentation produces multiple interwoven effects: reductions of biodiversity between 13% and 75%, decreasing forest biomass, and changes in nutrient cycling^6^. The effects of fragmentation are not only important from an ecological point of view but also that of human activities, as ecosystem services are deeply influenced by the level of landscape fragmentation^7–9^.

Ecosystems harbour hundreds of populations interacting through complex networks and present feedbacks at different levels of organization^10,11^, external forcings can produce abrupt changes from one state to another, called critical transitions^12^. Complex systems can experience two general classes of critical transitions^13^. In so-called first-order transitions, a catastrophic regime shift that is mostly irreversible occurs because of the existence of alternative stable states^14^. This class of transitions is suspected to be present in a variety of ecosystems such as lakes, woodlands, coral reefs^14^, semi-arid grasslands^15^, and fish populations^16^. They can be the result of positive feedback mechanisms^17^; for example, fires in some forest ecosystems were more likely to occur in previously burned areas than in unburned places^18^.

The other class of critical transitions are second order transitions^19^. In these cases, there is a narrow region where the system suddenly changes from one domain to another in a continuous and reversible way. Such transitions have been suggested for tropical forests^20,21^, semi-arid mountain ecosystems^22^, and tundra shrublands^23^. The transition happens at a critical point where we can observe scale-invariant fractal structures characterized by power law patch distributions^24^.

The spatial phenomena observed in continuous critical transitions are related to connectivity, a fundamental property of general systems and ecosystems from forests^25^ to marine ecosystems^26^ and the whole biosphere^27^. When a system goes from a fragmented to a connected state we say that it percolates^13^. Percolation implies that there is a path of connections that involve the whole system. Thus we can characterize two domains or phases: one dominated by short-range interactions which does not allow information (used in a broad sense, e.g. species dispersal or movement) to spread through the whole system, and another in which long-range interactions are possible and information can spread throughout the system. Thus, there is a critical “percolation threshold” between the two phases, and the system could be driven close to or beyond this point by an external force (called tunning parameter); climate change, deforestation and forest fire are the main forces that could be the drivers of such a phase change in contemporary forests^3,6^. There are several applications of this concept in ecology: species’ dispersal strategies are influenced by percolation thresholds in three-dimensional forest structure^28^, and it has been shown that species distributions also have percolation thresholds^29^. This implies that pushing the system below the percolation threshold could produce a biodiversity collapse^30–32^; conversely, being in a connected state (above the threshold) could accelerate the invasion of the forest into prairie^23,33^.

One of the main challenges with systems that can experience critical transitions–of any kind–is that the value of the critical threshold is not known in advance. In addition, because near the critical point a small change can precipitate a state shift in the system, they are difficult to predict. Several methods have been developed to detect if a system is close to the critical point, e.g. a deceleration in recovery from perturbations, or an increase in variance in the spatial or temporal pattern^34–37^.

The existence of a critical transition between two states has been established for forest at a global scale in different works^38–40^. It is generally believed that this constitutes a first order catastrophic transition. There are several processes that can convert a catastrophic transition to a second order transition^17^. These include stochasticity, such as demographic fluctuations, spatial heterogeneities, and/or dispersal limitation. All these components are present in forest around the globe^41–43^, and thus continuous transitions might be more probable than catastrophic transitions. Moreover, there is evidence of recovery in systems that supposedly suffered an irreversible transition produced by overgrazing^44,45^ and desertification^46^. From this basis, we applied indices derived from second-order transitions to global forest cover dynamics.

In this study, our objective is to look for evidence that forests around the globe are near continuous critical points that represent a fragmentation threshold. We use the framework of percolation to first evaluate if forest patch distribution at a continental scale is described by a power law distribution and then examine the fluctuations of the largest patch. The advantage of using data at a continental scale is that for very large systems the transitions are very sharp^13^ and thus much easier to detect than at smaller scales, where noise can mask the signals of the transition.

## Methods

### Study areas definition

We analysed mainland forests at a continental scale, covering the whole globe, by delimiting land areas with a near-contiguous forest cover, separated from each other by large non-forested areas. We defined three forest regions in America: South America temperate forest (SAT), subtropical and tropical forest up to Mexico (SAST), USA and Canada forest (NA). Europe and North Asia are one region (EUAS), then South-east Asia (SEAS), Africa (AF), and Australia (OC). We also analysed islands larger than 10^5^ km^2^. This criterion to delimit regions is based on percolation theory that assumes some kind of connectivity in the study area (Appendix Table S2, Figs S1–S6).

### Forest patch distribution

We studied forest patch distribution in each area from 2000 to 2015 using the MODerate-resolution Imaging Spectroradiometer (MODIS) Vegetation Continuous Fields (VCF) Tree Cover dataset version 051^47^. This dataset is produced at a global level with a 231-m resolution on an annual basis. There are several definitions of forest based on percent tree cover^48^ and these are used to convert the percentage tree cover to a binary image of forest and non-forest pixels; we chose a range from 20% to 40% threshold in 5% increments. This range is centred in the definition used by the United Nations’ International Geosphere-Biosphere Programme^49^, studies of global fragmentation^6^ and includes the range used in other studies of critical transitions^50^. Using this range we avoid the errors produced by low discrimination of MODIS VCF between forest and dense herbaceous vegetation at low forest cover and the saturation of MODIS VCF in dense forests^51^. We repeated all analyses across this set of thresholds, except in some specific cases described below. Patches of contiguous forest were determined in the binary image by grouping connected forest pixels using a neighbourhood of 8 forest units (Moore neighbourhood). The MODIS VCF product does not discriminate between tree types, and so besides natural forest it includes plantations of tree crops like rubber, oil palm, eucalyptus and other managed stands^52^. Even though datasets with lower resolutions than MODIS VCF, like MODIS Land Cover Type, have been used to study fragmentation^53^, products with higher resolution that describe forest cover also exist^54^. As we analyse the time series of forest patches, we cannot use the^54^ dataset which has a very limited temporal resolution (years 2000 & 2012).

### Percolation theory

A more in-depth introduction to percolation theory can be found elsewhere^24^ and a review from an ecological point of view is available^55^. Here, to explain the basic elements of percolation theory we formulate a simple model: we represent our area of interest by a square lattice and each site of the lattice can be occupied–e.g. by forest–with a probability *p*, thus the lattice will be more occupied when *p* is greater, but the sites are randomly distributed. We defined patches with the same 8 sites neighbourhood previously mentioned. When *p* is increased from low values, a patch that connects the whole lattice suddenly appears. At this point, it is said that the system percolates and the value of *p* is the critical point *p*_*c*_.

Thus percolation is characterized by two well-defined phases: the unconnected phase when *p* < *p*_*c*_, in which species cannot travel far inside the forest, as it is fragmented; in a general sense, information cannot spread. The second is the connected phase when *p* > *p*_*c*_, species can move inside a forest patch from side to side of the lattice, i.e. information can spread over the whole area. Near the critical point, several scaling laws arise: the structure of the patch that spans the area is fractal, the size distribution of the patches is power-law, and other quantities also follow power-law scaling^24^.

The value of the critical point *p*_*c*_ depends on the geometry of the lattice and on the definition of the neighbourhood, but other power-law exponents only depend on the lattice dimension. Close to the critical point, the distribution of patch sizes is:

1 $${n}_{s}({p}_{c})\propto {s}^{-\alpha }$$

where *n*_*s*_(*p*) is the number of patches of size *s*. The exponent *α* does not depend on the details of the model and it is called universal^24^. These scaling laws can be applied to landscape structures that are approximately random, or correlated over short distances^56^. In physics, this is called “isotropic percolation universality class”, and corresponds to an exponent *α* = 2.05495. If we observe that the patch size distribution has another exponent it will not belong to this universality class and other mechanisms should be invoked to explain it. Percolation can also be generated by models that have some kind of memory^57,58^: for example, a patch that has been exploited for many years will recover differently than a recently deforested forest patch. In this case, the system could belong to a different universality class, or in some cases, there is no universality, in which case the value of *α* will depend on the parameters and details of the model^59^.

To illustrate these concepts, we conducted simulations with a simple forest model with only two states: forest and non-forest. This type of model is called a “contact process” and was introduced for epidemics^60^ but has many applications in ecology^19,56^ (see supplementary data, gif animations).

### Patch size distributions

We fitted the empirical distribution of forest patches calculated for each of the percentage forest cover thresholds we defined. We fit four distributions using maximum likelihood^61,62^: power-law, power-law with exponential cut-off, log-normal, and exponential. We assumed that the patch size distribution is a continuous variable discretised by the remote sensing data acquisition procedure.

The power-law distribution requires a lower bound for its scaling behaviour that is estimated from the data by maximizing the Kolmogorov-Smirnov (KS) statistic between the empirical and fitted cumulative distribution functions^62^. For the log-normal model, we constrain the *μ* parameter to positive values, this parameter controls the mode of the distribution and when is negative most of the probability density of the distribution lies outside the range of the forest patch size data^63^.

To select the best model we calculated the corrected Akaike Information Criteria (*AIC*_*c*_) and Akaike weights for each model^64^. Akaike weights (*w*_*i*_) are the weight of evidence in favour of model *i* being the best model among the candidate set of *N* models. Additionally, we computed a likelihood ratio test^62,65^ of the power law model against the other distributions. We calculated bootstrapped 95% confidence intervals^66^ for the parameters of the best model, using the bias-corrected and accelerated (BCa) bootstrap^67^ with 10000 replications.

### Largest patch dynamics

The largest patch connects the highest number of sites in the area and has been used to indicate fragmentation^25,68^. The size of the largest patch *S*_*max*_ has been studied in relation to percolation phenomena^24,69,70^ but seldom used in ecological studies (but see^56^). When the system is in a connected state (*p* > *p*_*c*_) the landscape is almost insensitive to the loss of a small fraction of forest, but close to the critical point a minor loss can have important effects^19,55^, because at this point the largest patch will have a filamentary structure, i.e. extended forest areas will be connected by thin threads. Small losses can thus produce large fluctuations.

To evaluate the fragmentation of the forest the proportion of the largest patch against the total area can be calculated^71^. The total area of the regions we are considering (Appendix S4, Figs S1–S6) may not be the same as the total area that the forest could potentially occupy, and thus a more accurate way to evaluate the weight of *S*_*max*_ is to use the total forest area, which can be easily calculated by summing all the forest pixels. We calculate the proportion of the largest patch for each year, dividing *S*_*max*_ by the total forest area of the same year: $R{S}_{max}={S}_{max}/{\sum }_{i}\,{S}_{i}$. When the proportion *RS*_*max*_ is large (more than 60%) the largest patch contains most of the forest so there are fewer small forest patches and the system is probably in a connected phase. Conversely, when it is low (less than 20%), the system is probably in a fragmented phase^72^. To define if a region will be in a connected or unconnected state we used the *RS*_*max*_ of the highest (i.e., most conservative) threshold of 40%, that represents the most dense area of forest within our chosen range. We assume that there are two alternative states for the critical transition–the forest could be fragmented or unfragmented. If *RS*_*max*_ is a good indicator forest’s state, its distribution of frequencies should be bimodal^15^, so we apply the Hartigan’s dip test that measures departures from unimodality^73^.

To evaluate if the forest is near a critical transition, we calculate the fluctuations of the largest patch Δ*S*_*max*_ = *S*_*max*_(*t*) − 〈*S*_*max*_〉, using the same formula for *RS*_*max*_. To characterize fluctuations we fitted three empirical distributions: power-law, log-normal, and exponential, using the same methods described previously. We expect that large fluctuations near a critical point have heavy tails (log-normal or power-law) and that fluctuations far from a critical point have exponential tails, corresponding to Gaussian processes^74^. We also apply the likelihood ratio test explained previously^62,65^; if the p-values obtained to compare the best distribution against the others are not significant we can not decide which is the best model. We generated animated maps showing the fluctuations of the two largest patches at 30% threshold, to aid in the interpretations of the results.

A robust way to detect if the system is near a critical transition is to analyse the increase in variance of the density^75^–in our case ‘density’ is the total forest cover divided by the area. But the variance increase in density appears when the system is very close to the transition^59^, and thus practically it does not constitute an early warning indicator. An alternative is to analyse the variance of the fluctuations of the largest patch Δ*S*_*max*_: the maximum is attained at the critical point but a significant increase occurs well before the system reaches the critical point^59,72^. In addition, before the critical fragmentation, the skewness of the distribution of Δ*S*_*max*_ is negative, implying that fluctuations below the average are more frequent. We characterized the increase in the variance using quantile regression: if variance is increasing the slopes of upper or/and lower quartiles should be positive or negative.

Statistical analyses were performed using the GNU R version 3.3.0^76^, to fit patch size distributions we used the Python package powerlaw^77^. For quantile regressions, we used the R package quantreg^78^, and for image processing we used MATLAB r2015b. The “bwconncomp” MATLAB function, which implements a flood-fill algorithm, was used to identify individual patches from binary images. The complete source code for image processing, statistical analysis and patch size data are available at figshare 10.6084/m9.figshare.4263905.

## Results

**Figure 1:**
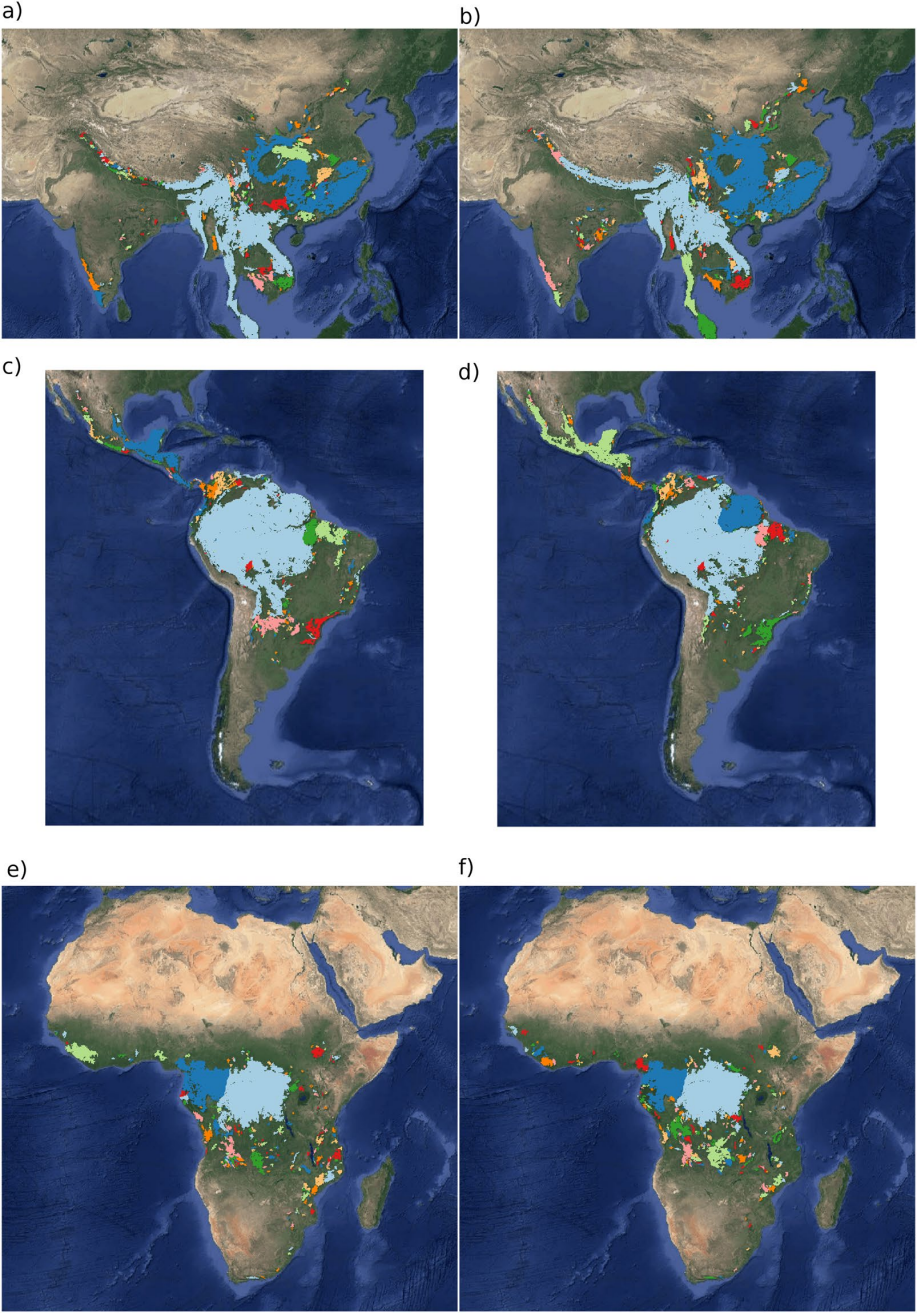
Forest patch distributions for continental regions for the years 2000 and 2014. The images are the 200 biggest patches, shown at a coarse pixel scale of 2.5 km. The regions are: (**a**,**b**) southeast Asia; (**c**,**d**) South America subtropical and tropical and (**e**,**f**) Africa mainland, for the years 2000 and 2014 respectively. The color palette was chosen to discriminate different patches and does not represent patch size. The imaged was composed with GIMP 2.8 software and the base maps retrieved from Google maps (Imagery(C)2018 NASA, TerraMetrics.

Figure 1 shows an example of the distribution of the biggest 200 patches for years 2000 and 2014. This distribution is highly variable; the biggest patch usually maintains its spatial location, but sometimes it breaks and then large temporal fluctuations in its size are observed, as we will analyse below. Smaller patches can merge or break more easily so they enter or leave the list of 200, and this is why there is a colour change across years.

**Figure 2:**
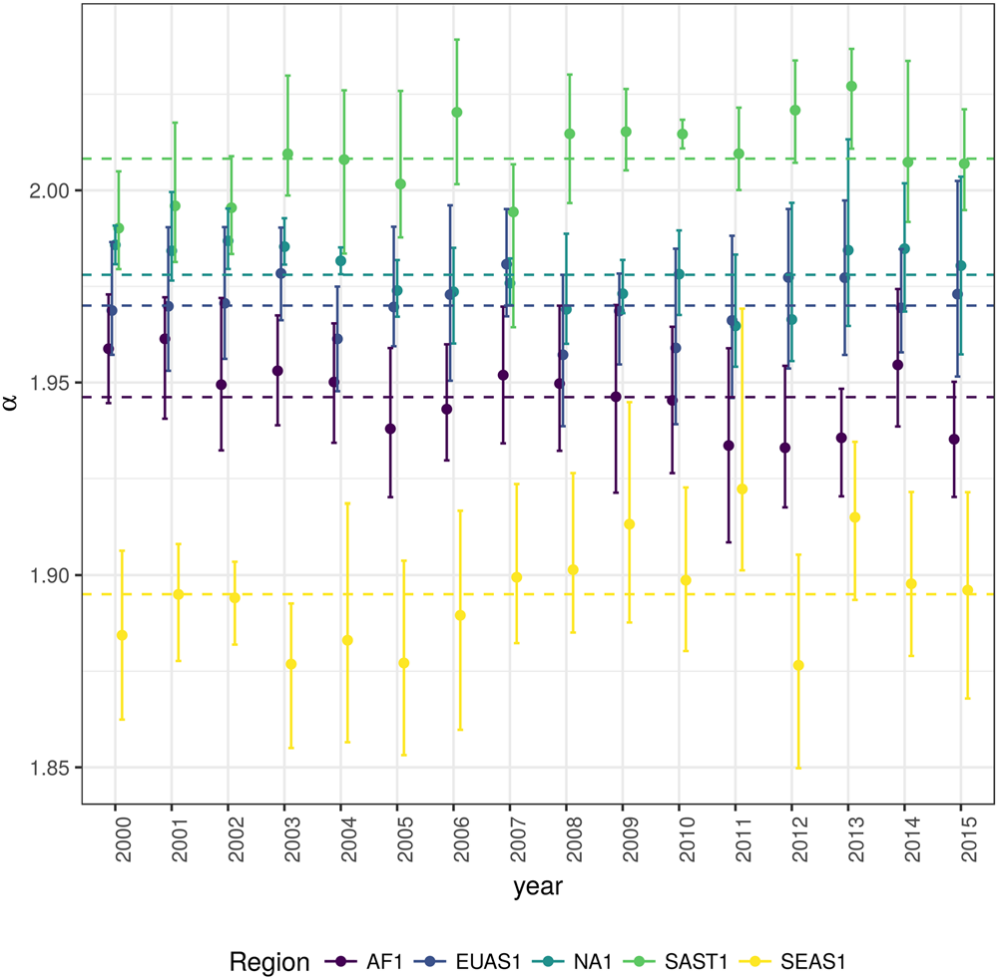
Power law exponents (*α*) of forest patch distributions for regions with total forest area >10^7^ km^2^. Dashed horizontal lines are the means by region, with 95% confidence interval error bars estimated by bootstrap resampling. The regions are AF1: Africa mainland, EUAS1: Eurasia mainland, NA1: North America mainland, SAST1: South America subtropical and tropical, SEAS1: Southeast Asia mainland.

The power law distribution was selected as the best model in 99% of the cases (Fig. S7). In a small number of cases (1%), the power law with exponential cutoff was selected, but the value of the parameter *α* was similar by ±0.03 to the pure power law (Table S1 and model fit data table), and thus we used the power law parameters for these cases (region EUAS3, SAST2) as well. In finite-size systems, the favoured model should be the power law with exponential cut-off because the power-law tails are truncated to the size of the system^24^. We observe that when the pure power-law model is the best model, the 64% of likelihood ratio tests against power law with exponential cutoff are not significant (p-value > 0.05). Instead, the likelihood ratio test clearly differentiates the power law model from the exponential model (100% cases p-value < 0.05), and the log-normal model (90% cases p-value < 0.05).

The global mean of the power-law exponent *α* is 1.967 and the bootstrapped 95% confidence interval is 1.964–1.970. The global values for each threshold are different, because their confidence intervals do not overlap, and their range goes from 1.90 to 2.01 (Table S1). Analysing the biggest regions (Fig. 2, Table S2) the northern hemisphere regions (EUAS1 & NA1) have similar values of *α* (1.97, 1.98), pantropical areas have different *α* with greatest values for South America (SAST1, 2.01) and in descending order Africa (AF1, 1.946) and Southeast Asia (SEAS1, 1.895). With greater *α* the fluctuations of patch sizes are lower and vice versa^79^.

We calculated the total forest areas and the largest patch *S*_*max*_ by year for different thresholds, and as expected these two values increase for smaller thresholds (Table S3). We expect fewer variations in the largest patch relative to total forest area *RS*_*max*_ (Fig. S9); in ten cases it stayed near or higher than 60% (EUAS2, NA5, OC2, OC3, OC4, OC5, OC6, OC8, SAST1, SAT1) over the 25–35 range or more. In four cases it stayed around 40% or less, at least over the 25–30% range (AF1, EUAS3, OC1, SAST2), and in six cases there is a crossover from more than 60% to around 40% or less (AF2, EUAS1, NA1, OC7, SEAS1, SEAS2). This confirms the criteria of using the most conservative threshold value of 40% to interpret *RS*_*max*_ with regard to the fragmentation state of the forest. The frequency of *RS*_*max*_ showed bimodality (Fig. S10) and the dip test rejected unimodality (D = 0.0416, p-value = 0.0003), which also implies that *RS*_*max*_ is a good index to study the fragmentation state of the forest.

**Figure 3:**
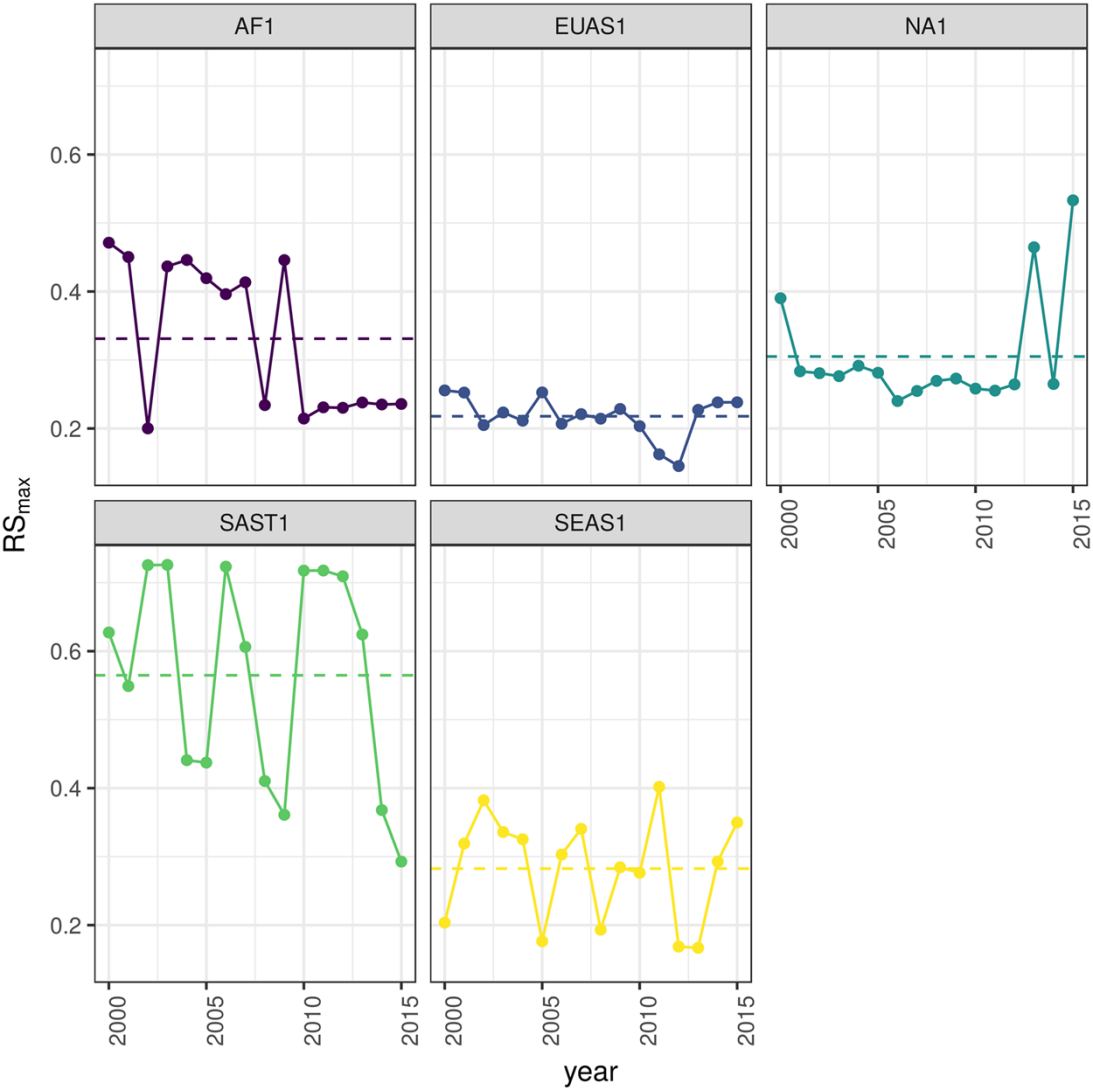
Largest patch proportion relative to total forest area *RS*_*max*_, for regions with total forest area >10^7^ km^2^. We show here the *RS*_*max*_ calculated using a threshold of 40% of forest in each pixel to determine patches. Dashed lines are averages across time. The regions are AF1: Africa mainland, EUAS1: Eurasia mainland, NA1: North America mainland, SAST1: South America tropical and subtropical, SEAS1: Southeast Asia mainland.

The *RS*_*max*_ for regions with more than 10^7^ km^2^ of forest is shown in Fig. 3. South America tropical and subtropical (SAST1) is the only region with an average close to 60%, the other regions are below 30%. Eurasia mainland (EUAS1) has the lowest value near 20%. For regions with less total forest area (Fig. S10, Table S3), Great Britain (EUAS3) has the lowest proportion less than 5%, Java (OC7) and Cuba (SAST2) are under 25%, while other regions such as New Guinea (OC2), Malaysia/Kalimantan (OC3), Sumatra (OC4), Sulawesi (OC5) and South New Zealand (OC6) have a very high proportion (75% or more). Philippines (SEAS2) seems to be a very interesting case because it seems to be under 30% until the year 2007, fluctuates around 30% in years 2008–2010, then jumps near 60% in 2011–2013 and then falls again to 30%, this seems an example of a transition from a fragmented state to an unfragmented one (Fig. S11).

The Likelihood ratio test was not significant for the distributions of largest patch fluctuations Δ*RS*_*max*_ and Δ*S*_*max*_. Thus we cannot determine with confidence which is the best distribution. In only one case was the distribution selected by the Akaike criteria confirmed as the correct model for relative and absolute fluctuations (Table S4).

The animations of the two largest patches (see supplementary data, largest patch gif animations) qualitatively shows the nature of fluctuations and whether the state of the forest is connected or not. If the largest patch is always the same patch over time, the forest is probably not fragmented; this happens for regions with *RS*_*max*_ of more than 40% such as AF2 (Madagascar), EUAS2 (Japan), NA5 (Newfoundland) and OC3 (Malaysia). In regions with *RS*_*max*_ between 40% and 30%, the identity of the largest patch could change or stay the same in time. For OC7 (Java) the largest patch changes and for AF1 (Africa mainland) it stays the same. Only for EUAS1 (Eurasia mainland), we observed that the two largest patches are always the same, implying that these two large patches are produced by a geographical accident but they have the same dynamics. The regions with *RS*_*max*_ less than 25% SAST2 (Cuba) and EUAS3 (Great Britain) have an always-changing largest patch reflecting their fragmented state. A transition is observed in SEAS2 (Philippines), with the identity of the largest patch first variable, and then constant after 2010.

**Figure 4:**
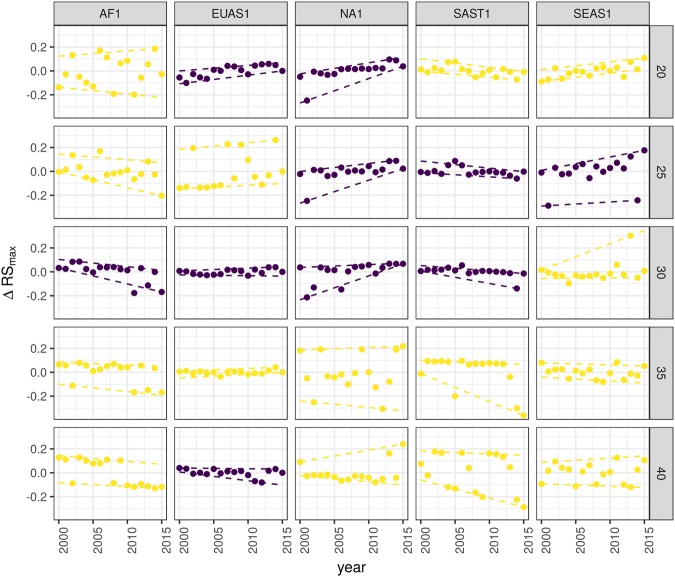
Largest patch fluctuations for regions with total forest area >10^7^ km^2^ across years. The patch sizes are relative to the total forest area of the same year. Dashed lines are 90% and 10% quantile regressions, to show if fluctuations were increasing; purple (dark) panels have significant slopes. The regions are AF1: Africa mainland, EUAS1: Eurasia mainland, NA1: North America mainland, SAST1: South America tropical and subtropical, SEAS1: Southeast Asia mainland.

The results of quantile regressions are almost identical for Δ*RS*_*max*_ and Δ*S*_*max*_ (Table S5). Among the biggest regions, Africa (AF1) has a similar pattern across thresholds but only the 30% threshold is significant; the upper and lower quantiles have significant negative slopes, but the lower quantile slope is lower, implying that negative fluctuations and variance are increasing (Fig. 4). Eurasia mainland (EUAS1) has significant slopes at 20%, 30% and 40% thresholds but the patterns are different at 20% variance is decreasing, at 30% and 40% only is increasing. Thus the variation of the most dense portion of the largest patch is increasing within a limited range. North America mainland (NA1) exhibits the same pattern at 20%, 25% and 30% thresholds: a significant lower quantile with positive slope, implying decreasing variance. South America tropical and subtropical (SAST1) have significant lower quantile with a negative slope at 25% and 30% thresholds indicating an increase in variance. SEAS1 has an upper quantile with a significant positive slope for 25% threshold, indicating an increasing variance. The other regions, with forest area smaller than 10^7^ km^2^ are shown in Fig. S11 and Table S5. For Philippines (SEAS2), the slopes of lower quantiles are positive for thresholds 20% and 25%, and the upper quantile slopes are positive for thresholds 30% and 40%; thus variance is decreasing at 20–25% and increasing at 30–40%.

The conditions that indicate that a region is near a critical fragmentation threshold are that patch size distributions follow a power law; variance of Δ*RS*_*max*_ is increasing in time, and skewness is negative. All these conditions must happen at the same time at least for one threshold. When the threshold is higher more dense regions of the forest are at risk. This happens for Africa mainland (AF1), Eurasia mainland(EUAS1), Japan (EUAS2), Australia mainland (OC1), Malaysia/Kalimantan (OC3), Sumatra (OC4), South America tropical & subtropical (SAST1), Cuba (SAST2), Southeast Asia, Mainland (SEAS1).

## Discussion

We found that the forest patch distribution of all regions of the world, spanning tropical rainforests, boreal and temperate forests, followed power laws through seven orders of magnitude. Power laws have previously been found for several kinds of vegetation, but never at global scales as in this study. Moreover, the range of the estimated power law exponents is relatively narrow (1.90–2.01), even though we used a range of different thresholds levels. This suggests the existence of one unifying mechanism that acts at continental scales, affecting forest spatial structure and dynamics.

A possible mechanism for the emergence of power laws in forests is isotropic percolation^20^: forest sites disappear at random positions when the density of forest is near the critical point, and thus the power law structures arise. This requires the tuning of an external environmental condition to carry the system to this point. We did not expect forest growth to be a random process at local scales, but it is possible that combinations of factors cancel out to produce seemingly random forest dynamics at large scales. This has been suggested as a mechanism for the observed power laws of global tropical forest in year 2000^21^. In this case, we should have observed power laws in a limited set of situations that coincide with a critical point, but instead, we observed pervasive power law distributions. Thus isotropic percolation does not seem to be the mechanism that produces the observed distributions.

Another possible mechanism is facilitation^80,81^: a patch surrounded by forest will have a smaller probability of being deforested or degraded than an isolated patch. The model of Scanlon *et al*.^82^ showed an *α* = 1.34 which is far from our results (1.90–2.01 range). Another model but with three states (tree/non-tree/degraded), including local facilitation and grazing, has also been used to obtain power laws patch distributions without external tuning and exhibited deviations from power laws at high grazing pressures^83^. The values of the power law exponent *α* for this model depend on the intensity of facilitation: if it is more intense the exponent is higher, but the maximal values they obtained are still lower than the ones we observed. Thus an exploration of the parameters of this model is needed to find if this is a plausible mechanism.

A combination of spatial and temporal indicators is more reliable for detecting critical transitions^84^. We combined five criteria to evaluate the closeness of the system to a fragmentation threshold. Two were spatial: the forest patch size distribution, and the proportion of the largest patch relative to total forest area *RS*_*max*_. The other three were the distribution of temporal fluctuations in the largest patch size, the trend in the variance, and the skewness of the fluctuations. One of them, the distribution of temporal fluctuations or Δ*RS*_*max*_, cannot be applied with our temporal resolution due to the difficulties of fitting and comparing heavy-tailed distributions. The combination of the remaining four gives us an increased degree of confidence about the system being close to a critical transition.

Although our results suggest the existence of a unifying mechanism, different factors could be acting in different regions and perhaps different models are needed. As a consequence, there might exist various critical fragmentation thresholds. As we did not elucidate the mechanism and the factors that might be most important for each region, we cannot determine the theoretical critical point, and this is why we tried to find signals of critical transitions without knowing the exact value of the critical fragmentation threshold.

South America tropical and subtropical (SAST1), Southeast Asia mainland (SEAS1) and Africa mainland (AF1) met all criteria at least for one threshold; these regions generally experience the biggest rates of deforestation with a significant increase in loss of forest^54^. The most critical regions are Southeast Asia and Africa, because the proportion of the largest patch relative to total forest area *RS*_*max*_ was around 30% thus they are in a fragmented state. Due to our criteria for defining regions, we could not detect the effect of conservation policies applied at a country level, e.g. the Natural Forest Conservation Program in China, which has produced a 1.6% increase in forest cover and net primary productivity over the last 20 years^85^. Tropical South America with its high *RS*_*max*_ is also endangered but probably in an unfragmented state. Indonesia and Malaysia (OC3) have both high deforestation rates^54^; Sumatra (OC4) is the biggest island of Indonesia and where most deforestation occurs. Both regions show a high *RS*_*max*_ greater than 60%, and thus the forest is in an unfragmented state, but they met all other criteria, meaning that they are approaching a transition if current deforestation rates continue.

The Eurasian mainland region (EUAS1) is an extensive area with mainly temperate and boreal forest and a combination of forest loss due to fire^86^ and forestry. Russia, the biggest country, has experienced the largest rate of forest loss of all countries, but in the zone of coniferous forest, the largest gain is observed due to agricultural abandonment^87^. The loss is maximum at the most dense areas of forest^54^, and this coincides with our analysis that detected an increased risk at denser forest. This region also has a relatively low *RS*_*max*_ which means that it is probably near a fragmented state. A possible explanation of this is that in Russia after the collapse of the Soviet Union harvest was lower due to agricultural abandonment, but illegal overharvesting of high valued stands has increased in recent decades^88^. A region that is similar in forest composition to EAUS1 is North America (NA1); the two main countries involved, United States and Canada, have forest dynamics mainly influenced by fire and forestry, with both regions extensively managed for industrial wood production. North America has a higher *RS*_*max*_ than Eurasia and a positive skewness that excludes it from being near a critical transition.

The analysis of *RS*_*max*_ reveals that the island of Philippines (SEAS2) seems to be an example of a critical transition from an unconnected to a connected state, i.e. from a state with high fluctuations and low *RS*_*max*_ to a state with low fluctuations and high *RS*_*max*_. If we observe this pattern backwards in time, the decrease in variance become an increase, and negative skewness is constant, and thus the region exhibits the criteria of a critical transition (Table 1, Fig. S12). The actual pattern of transition to an unfragmented state could be the result of an active intervention by the government promoting conservation and rehabilitation of forests^89^. This confirms that the early warning indicators proposed here work in the correct direction. An important caveat is that the MODIS dataset does not detect if native forest is replaced by agroindustrial tree plantations like oil palms, which are among the main drivers of deforestation in this area^90^, for example in Indonesia and Malaysia^91^ (Regions OC2,OC3, OC4, OC5, OC7). This overestimates *RS*_*max*_ and in consequence, we underestimate the fragmentation risks of these areas. To improve the estimation of forest patches the Hansen’s Landsat derived dataset^54^ should be produced on a yearly basis. In addition, it would be important from a conservation point of view to develop specific algorithms to detect particular forest plantation types for each region–for example, combining high-resolution images (e.g. QuickBird 0.5 m) with change-detection of Landsat images^91,92^ to locate palm oil plantations.

**Table 1:** Regions and indicators of closeness to a critical fragmentation point.

Region	Description	*RS*_*max*_	Threshold	Variance of Δ*RS*_*max*_	Skewness
AF1	Africa mainland	0.33	30	Increase	−1.4653
AF2	Madagascar	0.48	20	Increase	−0.4461
EUAS1	Eurasia, mainland	0.22	20	Decrease	−0.5015
EUAS1			30	Increase	0.3113
EUAS1			40	Increase	−1.316
EUAS2	Japan	0.94	35	Increase	−0.3913
EUAS2			40	Increase	−0.5030
EUAS3	Great Britain	0.03	40	NS	0.1860
NA1	North America, mainland	0.31	20	Decrease	−2.2895
NA1			25	Decrease	−2.4465
NA1			30	Decrease	−1.6340
NA5	Newfoundland	0.54	40	NS	−0.1053
OC1	Australia, Mainland	0.36	30	Increase	0.0920
OC1			35	Increase	−0.8033
OC2	New Guinea	0.96	25	Decrease	−0.1003
OC2			30	Decrease	0.1214
OC2			35	Decrease	−0.0124
OC3	Malaysia/Kalimantan	0.92	35	Increase	−1.0147
OC3			40	Increase	−1.5649
OC4	Sumatra	0.84	20	Increase	−1.3846
OC4			25	Increase	−0.5887
OC4			30	Increase	−1.4226
OC5	Sulawesi	0.82	40	NS	0.0323
OC6	New Zealand South Island	0.75	40	Increase	0.3024
OC7	Java	0.16	40	NS	2.0105
OC8	New Zealand North Island	0.64	40	NS	1.3175
SAST1	South America, Tropical and Subtropical forest	0.56	25	Increase	1.0519
SAST1			30	Increase	−2.7216
SAST2	Cuba	0.15	20	Increase	0.5049
SAST2			25	Increase	1.7263
SAST2			30	Increase	0.1665
SAST2			40	Increase	−0.5401
SAT1	South America, Temperate forest	0.54	25	Decrease	0.1483
SAT1			30	Decrease	−1.6059
SAT1			35	Decrease	−1.3809
SEAS1	Southeast Asia, Mainland	0.28	25	Increase	−1.3328
SEAS2	Philippines	0.33	20	Decrease	−1.6373
SEAS2			25	Decrease	−0.6648
SEAS2			30	Increase	0.1517
SEAS2			40	Increase	1.5996

Where: *RS*_*max*_ is the largest patch divided by the total forest area; Threshold is the value used to calculate patches from the MODIS VCF pixels; Δ*RS*_*max*_ are the fluctuations of *RS*_*max*_ around the mean and the increase or decrease in the variance was estimated using quantile regressions; skewness was calculated for *RS*_*max*_. NS means the results were non-significant. The conditions that determine the closeness to a fragmentation point are: increasing variance of Δ*RS*_*max*_ and negative skewness. *RS*_*max*_ indicates if the forest is unfragmented (>0.6) or fragmented (<0.3).

Deforestation and fragmentation are closely related. At low levels of habitat reduction species population will decline proportionally; this can happen even when the habitat fragments retain connectivity. As habitat reduction continues, the critical threshold is approached and connectivity will have large fluctuations^93^. This could trigger several negative synergistic effects: population fluctuations and the possibility of extinctions will rise, increasing patch isolation and decreasing connectivity^93^. This positive feedback mechanism will be enhanced when the fragmentation threshold is reached, resulting in the loss of most habitat specialist species at a landscape scale^30^. If a forest is already in a fragmented state, a second critical transition from forest to non-forest could happen: the desertification transition^59^. Considering the actual trends of habitat loss, and studying the dynamics of non-forest patches–instead of the forest patches–the risk of this kind of transition could be estimated. The simple models proposed previously could also be used to estimate if these thresholds are likely to be continuous and reversible or discontinuous and often irreversible^94^, and the degree of protection (e.g. using the set-asides strategy^95^) that would be necessary to stop this trend.

Therefore, even if critical thresholds are reached only in some forest regions at a continental scale, a cascading effect with global consequences could still be produced^96^. The risk of such an event will be higher if the dynamics of separate continental regions are coupled^27^. At least three of the regions defined here are considered tipping elements of the earth climate system that could be triggered during this century^97^. These were defined as policy-relevant tipping elements so that political decisions could determine whether the critical value is reached or not. Thus the criteria proposed here could be used as a more sensitive system to evaluate the closeness of a tipping point at a continental scale. Further improvements will produce quantitative predictions about the temporal horizon where these critical transitions could produce significant changes in the studied systems.

## Data Accessibility

The MODIS VCF product is freely available from NASA at https://search.earthdata.nasa.gov/. Csv text file with model fits for patch size distribution, and model selection for all the regions; Gif Animations of a forest model percolation; Gif animations of largest patches; patch size files for all years and regions used here; and all the R, Python and Matlab scripts are available at figshare 10.6084/m9.figshare.4263905.

## Electronic supplementary material

Supplementary information

## Data Availability

All the raw data is from public repositories, the data supporting the results is archived at figshare public repository and the data DOI is included at in the article.
