# Supplementary material for: Power laws and critical fragmentation in global forests
Source: Sci Rep. 2018 Dec 10;8:17766. doi: 10.1038/s41598-018-36120-w (PMC6288094; doi:10.1038/s41598-018-36120-w)
Supplement: Supplementary file 1 — Supplementary information [file 41598_2018_36120_MOESM1_ESM.pdf]

Supplementary Information

Power laws and critical fragmentation in global forests

Leonardo A. Saravia, **Santiago R. Doyle**, **Ben Bond-Lamberty**

Contact model description

The area of interest by a square lattice and each site of the lattice can be occupied by forest or empty. A site with forest can become extinct with probability e, and produce another forest site in a neighborhood with probability c. We use a neighborhood defined by an isotropic power law probability distribution. We defined a single control parameter as $\lambda = c/e$ and ran simulations for the unfragmented state $\lambda < \lambda_c$, with $\lambda = 2$, near the critical point for $\lambda = 2.5$, and for the fragmented state with $\lambda = 5$. We generated gif animations with these 3 cases to compare with the animations from real forest patches.

Metadata for files available at figshare

Model fits: "Fitted_Patch_models.csv" and "Fitted_Patch_models.txt" are Csv and tab separated text files with model fits for patch size distribution, and model selection for all the regions. We used the the Akaike criterion to select the best model, and a likelihood ratio test of the power law model against the other distributions. The models were fitted using maximum likelihood and the optimum Xmin was estimated from data usin Kolmogorov-Smirnov distance. The regions are: AF1 Africa Mainland; AF2 Madagascar; EUAS1 Eurasia mainland; EUAS2 Japan; EUAS3 Great Britain; NA1 North America mainland; NA5 Newfoundland; OC1 Australia mainland; OC2 New Guinea; OC3 Malaysia/Kalimantan; OC4 Sumatra; OC5 Sulawesi; OC6 New Zealand south island; OC7 Java; OC8 New Zealand north island; SAST1 South America, Tropical and subtropical forest up to Mexico; SAST2 Cuba; SAT1 South America Temperate forest; SEAS1 Southeast Asia mainland; SEAS2 Philippines The fields are: Xmin the minimun value for the power law distribution; par1 & par2 are the parameters of the distributions Power law and exponential have only one parameter, Lognormal and Power law with exponential cutoff have two parameters; n is the number of observations used in the fitting process; LL is the log likelihood; AICc is the Akaike criterion corrected for small n; delta_AICc is the difference with the lowest AICc; AICc_weight are the Akaike weights; LRT and pvalue are the likelihood ratio test with its p-value Available at figshare http://dx.doi.org/10.6084/m9.figshare.426390A

Gif Animations of a forest model percolation: These are animations showing the subcritical, critical, and super critical states. We use a a simple contact process in which an occupied site (green) represent forest. The largest forest patch is represented in black and empty sites are white. The simulations were made with a lattice of 256x256, the exponent of the power law dispersal kernel was 2.18, and $\lambda = c/e$ has the following values, 2 for the subcritical state, 2.5 near the critical state, and 5 for the supercritical state. File PercolationAnimations.zip, available at figshare http://dx.doi.org/10.6084/m9.figshare.4263905

Gif Animations of largest patches: These show the temporal dynamics of the two largest patches for all the regions studied at a coarse resolution (2 km pixel). File **LargestPatchAnimations.zip**, available at figshare http://dx.doi.org/10.6084/m9.figshare.4263905

Tables

Table S1: Mean power-law exponent and Bootstrapped 95% confidence intervals by threshold.

threshold	mean α	Low CI	High CI
20	1.9995	1.9916	2.0085
25	1.9637	1.9564	1.9710
30	1.9383	1.9321	1.9448
35	1.9164	1.9104	1.9223
40	1.9002	1.8949	1.9059

Table S2: Mean power-law exponent and Bootstrapped 95% confidence intervals across thresholds by region and year. The regions are: AF1 Africa Mainland; AF2 Madagascar; EUAS1 Eurasia mainland; EUAS2 Japan; EUAS3 Great Britain; NA1 North America mainland; NA5 Newfoundland; OC1 Australia mainland; OC2 New Guinea; OC3 Malaysia/Kalimantan; OC4 Sumatra; OC5 Sulawesi; OC6 New Zealand south island; OC7 Java; OC8 New Zealand north island; SAST1 South America, Tropical and subtropical forest up to Mexico; SAST2 Cuba; SAT1 South America Temperate forest; SEAS1 Southeast Asia mainland; SEAS2 Philippines

Region	year	mean α	Low CI	High CI
AF1	2000	1.9589	1.9446	1.9729
AF1	2001	1.9613	1.9406	1.9722
AF1	2002	1.9493	1.9323	1.9730
AF1	2003	1.9532	1.9389	1.9675
AF1	2004	1.9499	1.9346	1.9654
AF1	2005	1.9380	1.9210	1.9590
AF1	2006	1.9431	1.9297	1.9588
AF1	2007	1.9518	1.9342	1.9697
AF1	2008	1.9498	1.9322	1.9719
AF1	2009	1.9465	1.9213	1.9713
AF1	2010	1.9456	1.9264	1.9645
AF1	2011	1.9339	1.9085	1.9588
AF1	2012	1.9328	1.9178	1.9543
AF1	2013	1.9357	1.9204	1.9484
AF1	2014	1.9546	1.9386	1.9743
AF1	2015	1.9351	1.9202	1.9502
AF2	2000	1.9091	1.8994	1.9162
AF2	2001	1.9068	1.8931	1.9320
AF2	2002	1.9023	1.8874	1.9271
AF2	2003	1.9167	1.9051	1.9259
AF2	2004	1.9150	1.9032	1.9351
AF2	2005	1.9070	1.8956	1.9295
AF2	2006	1.9047	1.8843	1.9255
AF2	2007	1.8997	1.8924	1.9077
AF2	2008	1.9074	1.8824	1.9414
AF2	2009	1.9196	1.9149	1.9248
AF2	2010	1.9177	1.8868	1.9456
AF2	2011	1.9335	1.8920	1.9630
AF2	2012	1.9163	1.8920	1.9290
AF2	2013	1.9446	1.9343	1.9573
AF2	2014	1.9660	1.9475	1.9856
AF2	2015	1.9445	1.9336	1.9549
EUAS1	2000	1.9688	1.9572	1.9864
EUAS1	2001	1.9696	1.9530	1.9904
EUAS1	2002	1.9707	1.9561	1.9893
EUAS1	2003	1.9785	1.9662	1.9906
EUAS1	2004	1.9612	1.9477	1.9748
EUAS1	2005	1.9697	1.9594	1.9905
EUAS1	2006	1.9727	1.9505	1.9961
EUAS1	2007	1.9809	1.9672	1.9951
EUAS1	2008	1.9574	1.9386	1.9780
EUAS1	2009	1.9686	1.9547	1.9784
EUAS1	2010	1.9590	1.9399	1.9843
EUAS1	2011	1.9661	1.9444	1.9882
EUAS1	2012	1.9771	1.9536	1.9951

Region	year	mean α	Low CI	High CI
EUAS1	2013	1.9774	1.9561	1.9974
EUAS1	2014	1.9694	1.9578	1.9847
EUAS1	2015	1.9730	1.9515	2.0024
EUAS2	2000	1.9195	1.8855	1.9639
EUAS2	2001	1.9036	1.8745	1.9363
EUAS2	2002	1.9133	1.8777	1.9495
EUAS2	2003	1.9548	1.9442	1.9738
EUAS2	2004	1.8876	1.8460	1.9548
EUAS2	2005	1.9038	1.8746	1.9418
EUAS2	2006	1.9314	1.8974	1.9602
EUAS2	2007	1.8989	1.8403	1.9423
EUAS2	2008	1.9149	1.8912	1.9345
EUAS2	2009	1.9078	1.8752	1.9604
EUAS2	2010	1.9011	1.8533	1.9888
EUAS2	2011	1.9182	1.8741	1.9737
EUAS2	2012	1.8962	1.8622	1.9503
EUAS2	2013	1.9012	1.8579	1.9398
EUAS2	2014	1.9154	1.8849	1.9690
EUAS2	2015	1.8944	1.8653	1.9479
EUAS3	2000	2.0289	2.0078	2.0530
EUAS3	2001	2.0601	2.0231	2.1252
EUAS3	2002	2.0084	1.9870	2.0271
EUAS3	2003	1.9867	1.9690	2.0044
EUAS3	2004	2.0150	1.9760	2.1155
EUAS3	2005	1.9994	1.9781	2.0321
EUAS3	2006	2.0099	1.9705	2.0470
EUAS3	2007	1.9686	1.9618	1.9739
EUAS3	2008	2.0438	2.0068	2.1112
EUAS3	2009	2.0207	1.9962	2.0328
EUAS3	2010	2.0133	1.9888	2.0354
EUAS3	2011	1.9934	1.9294	2.0628
EUAS3	2012	1.9810	1.9587	2.0035
EUAS3	2013	2.0117	1.9677	2.0502
EUAS3	2014	1.9818	1.9617	1.9989
EUAS3	2015	2.0434	1.9931	2.0858
NA1	2000	1.9858	1.9809	1.9913
NA1	2001	1.9843	1.9765	1.9982
NA1	2002	1.9868	1.9795	1.9953
NA1	2003	1.9853	1.9807	1.9927
NA1	2004	1.9817	1.9783	1.9851
NA1	2005	1.9739	1.9671	1.9819
NA1	2006	1.9736	1.9601	1.9850
NA1	2007	1.9759	1.9702	1.9823
NA1	2008	1.9689	1.9601	1.9894
NA1	2009	1.9731	1.9680	1.9814
NA1	2010	1.9781	1.9676	1.9904
NA1	2011	1.9647	1.9541	1.9833
NA1	2012	1.9665	1.9555	1.9968
NA1	2013	1.9846	1.9653	2.0133
NA1	2014	1.9847	1.9684	2.0018
NA1	2015	1.9802	1.9573	2.0036
NA5	2000	2.0880	1.9856	2.1494

Region	year	mean α	Low CI	High CI
NA5	2001	2.0140	1.9695	2.0795
NA5	2002	2.0478	1.9725	2.1013
NA5	2003	2.0269	1.9429	2.1115
NA5	2004	1.9726	1.9296	2.0410
NA5	2005	2.0363	1.9418	2.1166
NA5	2006	2.0645	1.9803	2.1415
NA5	2007	2.0727	1.9726	2.1849
NA5	2008	2.0901	2.0122	2.2129
NA5	2009	2.0718	1.9897	2.1577
NA5	2010	2.1502	2.0204	2.2527
NA5	2011	2.0167	1.9392	2.0945
NA5	2012	2.1371	2.0544	2.2295
NA5	2013	2.1277	2.0201	2.2598
NA5	2014	2.0359	1.9306	2.1654
NA5	2015	2.0497	1.9929	2.1103
OC1	2000	1.8626	1.8530	1.8729
OC1	2001	1.8817	1.8720	1.8944
OC1	2002	1.9033	1.8966	1.9109
OC1	2003	1.8665	1.8588	1.8775
OC1	2004	1.8477	1.8315	1.8651
OC1	2005	1.8608	1.8523	1.8709
OC1	2006	1.8657	1.8505	1.8812
OC1	2007	1.8796	1.8630	1.8940
OC1	2008	1.8710	1.8568	1.8922
OC1	2009	1.8942	1.8838	1.9042
OC1	2010	1.8781	1.8641	1.8931
OC1	2011	1.8470	1.8388	1.8545
OC1	2012	1.8842	1.8702	1.8996
OC1	2013	1.9218	1.9114	1.9341
OC1	2014	1.9450	1.9397	1.9502
OC1	2015	1.9015	1.8865	1.9153
OC2	2000	1.9466	1.9299	1.9604
OC2	2001	2.0319	1.9964	2.0802
OC2	2002	1.9990	1.9721	2.0348
OC2	2003	2.0386	1.9793	2.0955
OC2	2004	1.9839	1.9530	2.0277
OC2	2005	2.0630	1.9896	2.1194
OC2	2006	1.9766	1.9372	2.0214
OC2	2007	1.9517	1.9223	1.9839
OC2	2008	1.9396	1.9154	1.9691
OC2	2009	1.9969	1.9727	2.0321
OC2	2010	1.9582	1.9350	1.9902
OC2	2011	1.9979	1.9522	2.0628
OC2	2012	2.0074	1.9753	2.0416
OC2	2013	2.0125	1.9717	2.0538
OC2	2014	2.0244	1.9849	2.0671
OC2	2015	2.0011	1.9613	2.0591
OC3	2000	1.8652	1.8503	1.8858
OC3	2001	1.9502	1.9194	1.9825
OC3	2002	1.9469	1.9407	1.9532
OC3	2003	1.9927	1.9485	2.0371
OC3	2004	1.9277	1.9180	1.9366

Region	year	mean α	Low CI	High CI
OC3	2005	1.9675	1.9279	2.0145
OC3	2006	1.9244	1.8988	1.9513
OC3	2007	1.8855	1.8646	1.9106
OC3	2008	1.9363	1.8989	1.9910
OC3	2009	1.9166	1.8868	1.9486
OC3	2010	1.9701	1.9199	2.0346
OC3	2011	1.9703	1.9318	2.0267
OC3	2012	1.9191	1.8943	1.9456
OC3	2013	1.9062	1.8750	1.9593
OC3	2014	1.9849	1.9481	2.0289
OC3	2015	1.9253	1.8890	1.9746
OC4	2000	1.8977	1.8735	1.9174
OC4	2001	1.9209	1.8938	1.9412
OC4	2002	1.9501	1.9272	1.9732
OC4	2003	1.9147	1.9018	1.9298
OC4	2004	1.9011	1.8746	1.9217
OC4	2005	1.9333	1.8983	1.9604
OC4	2006	1.8973	1.8930	1.9013
OC4	2007	1.9237	1.8927	1.9487
OC4	2008	1.9037	1.8863	1.9328
OC4	2009	1.9195	1.8953	1.9580
OC4	2010	1.9242	1.9046	1.9562
OC4	2011	1.9373	1.9156	1.9658
OC4	2012	1.9547	1.9133	1.9811
OC4	2013	1.9169	1.8822	1.9459
OC4	2014	1.9476	1.9185	1.9886
OC4	2015	1.9042	1.8804	1.9225
OC5	2000	1.9735	1.9455	2.0039
OC5	2001	1.9837	1.9491	2.0341
OC5	2002	1.9893	1.9741	2.0075
OC5	2003	2.0343	2.0028	2.0848
OC5	2004	2.0183	1.9535	2.0708
OC5	2005	1.9949	1.9589	2.0263
OC5	2006	1.9482	1.8960	1.9984
OC5	2007	2.0205	1.9720	2.0546
OC5	2008	1.9997	1.9409	2.0570
OC5	2009	2.0128	1.9716	2.0542
OC5	2010	2.0981	2.0480	2.1321
OC5	2011	2.0993	2.0283	2.1499
OC5	2012	2.0453	1.9966	2.0917
OC5	2013	2.0059	1.9477	2.0659
OC5	2014	2.0022	1.9470	2.0758
OC5	2015	1.9968	1.9444	2.0325
OC6	2000	1.8957	1.8839	1.9065
OC6	2001	1.9104	1.8903	1.9208
OC6	2002	1.9120	1.8906	1.9361
OC6	2003	1.9138	1.9048	1.9227
OC6	2004	1.9417	1.9245	1.9636
OC6	2005	1.9456	1.9191	1.9876
OC6	2006	1.9098	1.9012	1.9171
OC6	2007	1.9010	1.8713	1.9178
OC6	2008	1.9252	1.9039	1.9536

Region	year	mean α	Low CI	High CI
OC6	2009	1.9066	1.8895	1.9284
OC6	2010	1.9050	1.8971	1.9115
OC6	2011	1.9387	1.9273	1.9527
OC6	2012	1.8996	1.8889	1.9117
OC6	2013	1.9041	1.8866	1.9259
OC6	2014	1.9121	1.8939	1.9251
OC6	2015	1.9241	1.9046	1.9508
OC7	2000	1.9020	1.8650	1.9342
OC7	2001	1.8886	1.8568	1.9234
OC7	2002	1.8932	1.8662	1.9160
OC7	2003	1.8903	1.8614	1.9188
OC7	2004	1.8866	1.8448	1.9288
OC7	2005	1.8865	1.8493	1.9241
OC7	2006	1.8902	1.8707	1.9115
OC7	2007	1.8893	1.8469	1.9415
OC7	2008	1.8698	1.8593	1.8797
OC7	2009	1.9083	1.8615	1.9642
OC7	2010	1.9846	1.9523	2.0195
OC7	2011	1.9125	1.8714	1.9622
OC7	2012	1.8819	1.8420	1.9364
OC7	2013	1.8835	1.8556	1.9151
OC7	2014	1.9424	1.8941	1.9817
OC7	2015	1.8990	1.8469	1.9711
OC8	2000	1.9679	1.9219	2.0123
OC8	2001	1.9616	1.9432	1.9919
OC8	2002	1.9262	1.9157	1.9346
OC8	2003	2.0500	1.9850	2.1240
OC8	2004	1.9395	1.9171	1.9724
OC8	2005	2.0049	1.9531	2.0809
OC8	2006	2.0271	1.9891	2.0698
OC8	2007	1.9965	1.9475	2.0649
OC8	2008	2.0828	2.0389	2.1449
OC8	2009	2.0532	1.9990	2.1029
OC8	2010	1.9549	1.9122	1.9987
OC8	2011	2.0268	2.0044	2.0439
OC8	2012	1.9943	1.9788	2.0093
OC8	2013	2.0059	1.9748	2.0510
OC8	2014	2.0343	1.9967	2.0831
OC8	2015	1.9918	1.9495	2.0519
SAST1	2000	1.9902	1.9797	2.0052
SAST1	2001	1.9960	1.9813	2.0211
SAST1	2002	1.9955	1.9834	2.0088
SAST1	2003	2.0097	1.9987	2.0331
SAST1	2004	2.0078	1.9835	2.0260
SAST1	2005	2.0016	1.9878	2.0267
SAST1	2006	2.0205	2.0016	2.0392
SAST1	2007	1.9946	1.9624	2.0055
SAST1	2008	2.0145	1.9977	2.0301
SAST1	2009	2.0151	2.0052	2.0264
SAST1	2010	2.0147	2.0106	2.0184
SAST1	2011	2.0095	2.0006	2.0215
SAST1	2012	2.0209	2.0079	2.0338

Region	year	mean α	Low CI	High CI
SAST1	2013	2.0271	2.0095	2.0370
SAST1	2014	2.0074	1.9918	2.0303
SAST1	2015	2.0070	1.9949	2.0211
SAST2	2000	1.7960	1.7676	1.8321
SAST2	2001	1.8227	1.7823	1.8658
SAST2	2002	1.8171	1.7859	1.8721
SAST2	2003	1.8050	1.7757	1.8441
SAST2	2004	1.8332	1.8168	1.8485
SAST2	2005	1.7837	1.7668	1.8102
SAST2	2006	1.8694	1.8210	1.9255
SAST2	2007	1.8094	1.7722	1.8652
SAST2	2008	1.8316	1.8053	1.8508
SAST2	2009	1.8515	1.8189	1.8972
SAST2	2010	1.8366	1.8052	1.8772
SAST2	2011	1.8216	1.7870	1.8572
SAST2	2012	1.7817	1.7556	1.8200
SAST2	2013	1.8692	1.8149	1.9121
SAST2	2014	1.8602	1.8022	1.9494
SAST2	2015	1.8522	1.7932	1.9154
SAT1	2000	1.8649	1.8338	1.8886
SAT1	2001	1.8340	1.8203	1.8431
SAT1	2002	1.8444	1.8072	1.8836
SAT1	2003	1.8369	1.8325	1.8472
SAT1	2004	1.8342	1.7906	1.8768
SAT1	2005	1.8424	1.8140	1.8779
SAT1	2006	1.8166	1.7971	1.8317
SAT1	2007	1.8193	1.8083	1.8303
SAT1	2008	1.8055	1.7796	1.8343
SAT1	2009	1.8287	1.8171	1.8447
SAT1	2010	1.8047	1.7902	1.8299
SAT1	2011	1.8219	1.8058	1.8381
SAT1	2012	1.8327	1.8244	1.8403
SAT1	2013	1.8607	1.8535	1.8697
SAT1	2014	1.8478	1.8065	1.8885
SAT1	2015	1.7915	1.7728	1.8213
SEAS1	2000	1.8845	1.8624	1.9064
SEAS1	2001	1.8951	1.8777	1.9081
SEAS1	2002	1.8940	1.8819	1.9035
SEAS1	2003	1.8768	1.8560	1.8926
SEAS1	2004	1.8828	1.8565	1.9174
SEAS1	2005	1.8772	1.8536	1.9038
SEAS1	2006	1.8898	1.8573	1.9168
SEAS1	2007	1.8994	1.8823	1.9236
SEAS1	2008	1.9015	1.8851	1.9264
SEAS1	2009	1.9132	1.8877	1.9449
SEAS1	2010	1.8988	1.8803	1.9251
SEAS1	2011	1.9225	1.9012	1.9679
SEAS1	2012	1.8766	1.8482	1.9053
SEAS1	2013	1.9149	1.8965	1.9346
SEAS1	2014	1.8977	1.8790	1.9215
SEAS1	2015	1.8960	1.8674	1.9214
SEAS2	2000	1.8950	1.8182	1.9405

Region	year	mean α	Low CI	High CI
SEAS2	2001	1.9554	1.8822	2.0146
SEAS2	2002	1.8792	1.8310	1.9276
SEAS2	2003	1.9578	1.8913	2.0217
SEAS2	2004	1.9440	1.8880	1.9879
SEAS2	2005	1.9063	1.8616	1.9427
SEAS2	2006	1.9348	1.8923	1.9609
SEAS2	2007	1.9521	1.9129	1.9689
SEAS2	2008	1.9573	1.8939	1.9889
SEAS2	2009	1.9220	1.8609	1.9636
SEAS2	2010	1.9270	1.8939	1.9478
SEAS2	2011	1.9643	1.9350	1.9820
SEAS2	2012	1.9412	1.9206	1.9694
SEAS2	2013	1.8677	1.8233	1.8902
SEAS2	2014	1.9392	1.9010	1.9644
SEAS2	2015	1.9407	1.9005	1.9631

Table S3: Mean total patch area; largest patch S_{max} in Km^2; largest patch relative to total forest area RS_{max} and 95% bootstrapped confidence interval of RS_{max}, by region and thresholds, averaged across years. The regions are: AF1 Africa Mainland; AF2 Madagascar; EUAS1 Eurasia mainland; EUAS2 Japan; EUAS3 Great Britain; NA1 North America mainland; NA5 Newfoundland; OC1 Australia mainland; OC2 New Guinea; OC3 Malaysia/Kalimantan; OC4 Sumatra; OC5 Sulawesi; OC6 New Zealand south island; OC7 Java; OC8 New Zealand north island; SAST1 South America, Tropical and subtropical forest up to Mexico; SAST2 Cuba; SAT1 South America Temperate forest; SEAS1 Southeast Asia mainland; SEAS2 Philippines

Region	threshold	Average S_{max}	Total Patch Area	Average RS_{max}	Low CI	High CI
AF1	20	14804296.61	27261882	0.54	0.49	0.58
AF1	25	8427300.70	22448810	0.37	0.34	0.41
AF1	30	6603097.57	18876715	0.35	0.30	0.38
AF1	35	5859503.56	16232330	0.36	0.31	0.40
AF1	40	4760887.90	14387848	0.33	0.28	0.38
AF2	20	603361.45	821428	0.73	0.73	0.74
AF2	25	525221.23	704373	0.75	0.69	0.76
AF2	30	384084.00	595761	0.64	0.58	0.70
AF2	35	254644.77	502565	0.51	0.46	0.54
AF2	40	209648.53	436751	0.48	0.44	0.52
EUAS1	20	36898734	55387513	0.67	0.65	0.68
EUAS1	25	20027122	48063469	0.42	0.36	0.50
EUAS1	30	12268393	41951402	0.29	0.28	0.30
EUAS1	35	9666501	36725363	0.26	0.25	0.27
EUAS1	40	7005272	32094213	0.22	0.20	0.23
EUAS2	20	936893.72	983695	0.95	0.95	0.96
EUAS2	25	896632.59	943506	0.95	0.95	0.95
EUAS2	30	859718.16	906421	0.95	0.95	0.95
EUAS2	35	820994.90	868763	0.94	0.94	0.95
EUAS2	40	778313.14	829579	0.94	0.93	0.94
EUAS3	20	120068.82	446453	0.27	0.22	0.33
EUAS3	25	42035.81	331031	0.13	0.10	0.16
EUAS3	30	16244.21	247844	0.06	0.05	0.09
EUAS3	35	7651.45	185341	0.04	0.03	0.06
EUAS3	40	3918.11	139049	0.03	0.02	0.03
NA1	20	30508409.72	38139603	0.80	0.75	0.82
NA1	25	25970785.22	33749625	0.77	0.72	0.79
NA1	30	21226208.03	29730570	0.71	0.66	0.74
NA1	35	14520698.04	26016546	0.56	0.48	0.63
NA1	40	6909461.82	22563754	0.31	0.28	0.36
NA5	20	418826.73	432016	0.97	0.96	0.97
NA5	25	358724.36	381304	0.94	0.92	0.95
NA5	30	291364.28	332607	0.87	0.83	0.90
NA5	35	219408.70	283220	0.77	0.71	0.81
NA5	40	127791.32	231036	0.54	0.47	0.61
OC1	20	633410.80	2881701	0.22	0.21	0.24
OC1	25	565818.34	2300344	0.25	0.24	0.26
OC1	30	525313.35	1867240	0.28	0.27	0.29
OC1	35	491881.39	1534820	0.32	0.31	0.33
OC1	40	458177.75	1268736	0.36	0.35	0.37
OC2	20	3832485.22	3921625	0.98	0.98	0.98
OC2	25	3729540.57	3820224	0.98	0.97	0.98
OC2	30	3598986.08	3715287	0.97	0.95	0.97

Region	threshold	Average S_{max}	Total Patch Area	Average RS_{max}	Low CI	High CI
OC2	35	3479533.90	3604866	0.97	0.95	0.97
OC2	40	3355801.60	3497811	0.96	0.94	0.97
OC3	20	3450947.64	3513463	0.98	0.98	0.98
OC3	25	3300719.20	3386548	0.97	0.97	0.98
OC3	30	3142935.58	3255179	0.97	0.96	0.97
OC3	35	2971379.70	3117506	0.95	0.95	0.96
OC3	40	2747918.81	2978378	0.92	0.89	0.94
OC4	20	1852613.24	1923981	0.96	0.95	0.97
OC4	25	1699537.80	1813327	0.94	0.93	0.95
OC4	30	1556635.00	1698841	0.92	0.90	0.93
OC4	35	1395481.85	1582336	0.88	0.86	0.90
OC4	40	1234006.16	1468070	0.84	0.81	0.86
OC5	20	721115.01	783843	0.92	0.90	0.93
OC5	25	683484.30	749537	0.91	0.89	0.93
OC5	30	631344.12	715405	0.88	0.86	0.91
OC5	35	586142.65	683159	0.86	0.83	0.89
OC5	40	536133.57	653181	0.82	0.81	0.84
OC6	20	343263.55	412354	0.83	0.80	0.86
OC6	25	291228.48	371372	0.78	0.77	0.80
OC6	30	258446.45	339619	0.76	0.75	0.77
OC6	35	238376.80	316014	0.75	0.74	0.77
OC6	40	224887.77	298603	0.75	0.74	0.76
OC7	20	238558.68	386079	0.61	0.56	0.70
OC7	25	167000.23	313697	0.53	0.48	0.59
OC7	30	97558.04	254770	0.38	0.31	0.44
OC7	35	52191.42	210999	0.24	0.20	0.28
OC7	40	30672.31	179120	0.16	0.13	0.22
OC8	20	457707.05	506663	0.90	0.86	0.94
OC8	25	377814.69	457445	0.82	0.78	0.86
OC8	30	304062.74	408306	0.74	0.70	0.78
OC8	35	248573.15	362755	0.68	0.65	0.72
OC8	40	209320.66	324727	0.64	0.62	0.68
SAST1	20	37565778.84	53490576	0.70	0.68	0.72
SAST1	25	33340421.85	48749454	0.68	0.67	0.71
SAST1	30	30556744.07	44989122	0.68	0.65	0.69
SAST1	35	26493396.06	41993296	0.63	0.54	0.68
SAST1	40	22409091.19	39551845	0.56	0.49	0.63
SAST2	20	172890.38	351207	0.47	0.37	0.60
SAST2	25	89256.65	300405	0.28	0.22	0.39
SAST2	30	55348.94	259932	0.21	0.18	0.24
SAST2	35	40409.37	222703	0.18	0.16	0.21
SAST2	40	27621.52	186804	0.15	0.14	0.16
SAT1	20	782796.70	1180324	0.66	0.64	0.69
SAT1	25	673001.99	1067861	0.63	0.61	0.66
SAT1	30	587457.50	981545	0.60	0.58	0.61
SAT1	35	525323.54	909278	0.58	0.55	0.60
SAT1	40	460767.54	848650	0.54	0.51	0.57
SEAS1	20	15366420.54	20442364	0.75	0.74	0.76
SEAS1	25	12009720.52	17793031	0.67	0.60	0.71
SEAS1	30	6204306.06	15669303	0.40	0.37	0.47
SEAS1	35	4762660.23	13758367	0.35	0.32	0.37

Region	threshold	Average S_{max}	Total Patch Area	Average RS_{max}	Low CI	High CI
SEAS1	40	3402921.42	12018179	0.28	0.24	0.32
SEAS2	20	326831.68	397598	0.82	0.75	0.86
SEAS2	25	254349.14	358630	0.70	0.61	0.77
SEAS2	30	182400.51	326389	0.55	0.45	0.66
SEAS2	35	117537.97	296966	0.39	0.33	0.47
SEAS2	40	90903.67	269518	0.33	0.29	0.41

Table S4: Model selection for distributions of fluctuation of largest patch ΔS_{max} (Abs), and largest patch relative to total forest area ΔRS_{max} (Prop). The table shows for each region and threshold the best model selected by the Akaike criteria, the Akaike weight, the likelihod ration test statistic (LRT) against power law for Exponential and lognormal, and against exponential for power-law. The regions are: AF1, Africa Mainland, AF2, Madagascar; EUAS1 Eurasia mainland; EUAS2 Japan; EUAS3 Great Britain; NA1 North America mainland; NA5 Newfoundland; OC1 Australia mainland; OC2 New Guinea; OC3 Malaysia/Kalimantan; OC4 Sumatra; OC5 Sulawesi; OC6 New Zealand south island; OC7 Java; OC8 New Zealand north island; SAST1 South America, Tropical and subtropical forest up to Mexico; SAST2 Cuba; SAT1 South America, Temperate forest; SEAS1 Southeast Asia mainland; SEAS2 Philippines.

Region	threshold	model_name	par1	par2	X_{min}	n	LRT	p-value	AICc_weight	group
AF1	20	Exp	0.000	0.00	1294135.444	13	-2.599	0.072	0.793	Abs
AF1	20	Exp	14.089	0.00	0.048	13	-2.658	0.063	0.790	Prop
AF1	25	Exp	0.000	0.00	338476.964	12	-0.931	0.679	0.603	Abs
AF1	25	Exp	17.709	0.00	0.014	12	-1.365	0.548	0.679	Prop
AF1	30	Power	2.131	0.00	406099.045	12	0.062	0.974	0.441	Abs
AF1	30	Power	2.172	0.00	0.022	12	0.486	0.797	0.525	Prop
AF1	35	Power	3.270	0.00	892597.055	10	0.421	0.726	0.538	Abs
AF1	35	Power	3.014	0.00	0.052	10	0.072	0.946	0.466	Prop
AF1	40	Exp	0.000	0.00	1531828.156	8	-0.041	0.789	0.473	Abs
AF1	40	Exp	85.384	0.00	0.106	8	-0.220	0.160	0.486	Prop
AF2	20	Exp	0.000	0.00	4305.279	13	-1.685	0.469	0.735	Abs
AF2	20	Exp	56.036	0.00	0.005	13	-1.563	0.506	0.715	Prop
AF2	30	Exp	0.000	0.00	44026.281	12	-0.491	0.669	0.551	Abs
AF2	35	Exp	0.000	0.00	34477.590	12	-1.412	0.034	0.591	Abs
AF2	35	Exp	70.079	0.00	0.111	6	-0.045	0.839	0.491	Prop
AF2	40	Exp	0.000	0.00	21085.049	11	-1.213	0.330	0.684	Abs
EUAS1	20	Power	6.594	0.00	2798338.963	6	0.414	0.276	0.544	Abs
EUAS1	25	Power	4.534	0.00	5583402.353	10	0.520	0.500	0.529	Abs
EUAS1	25	Exp	19.021	0.00	0.111	10	-0.228	0.733	0.504	Prop
EUAS1	30	Exp	0.000	0.00	724333.122	9	-0.119	0.865	0.485	Abs
EUAS1	30	Exp	116.981	0.00	0.017	9	-0.316	0.664	0.529	Prop
EUAS1	35	Power	2.943	0.00	377476.319	7	0.673	0.485	0.612	Abs
EUAS1	35	Power	3.095	0.00	0.011	7	0.749	0.425	0.617	Prop
EUAS1	40	Exp	0.000	0.00	269997.687	11	-1.741	0.315	0.745	Abs
EUAS1	40	Exp	44.201	0.00	0.008	11	-1.632	0.354	0.729	Prop
EUAS2	20	Power	3.208	0.00	8333.329	9	0.114	0.899	0.480	Abs
EUAS2	20	Power	3.273	0.00	0.009	9	0.215	0.815	0.502	Prop
EUAS2	25	Power	5.364	0.00	14576.165	5	0.159	0.725	0.529	Abs
EUAS2	25	Power	5.066	0.00	0.015	5	0.037	0.932	0.500	Prop
EUAS2	30	Power	4.370	0.00	16128.279	5	0.127	0.834	0.522	Abs
EUAS2	30	Power	4.580	0.00	0.018	5	0.219	0.721	0.544	Prop
EUAS2	35	Exp	0.000	0.00	6367.329	12	-0.976	0.580	0.633	Abs
EUAS2	35	Power	2.430	0.00	0.011	10	0.079	0.962	0.465	Prop
EUAS2	40	Exp	0.000	0.00	8139.406	13	-0.967	0.636	0.636	Abs
EUAS2	40	Exp	47.311	0.00	0.010	13	-0.912	0.654	0.624	Prop
EUAS3	20	Power	3.749	0.00	44376.326	6	0.760	0.208	0.638	Abs
EUAS3	20	Power	4.552	0.00	0.109	6	0.805	0.145	0.654	Prop
EUAS3	25	Exp	0.000	0.00	16416.524	9	-0.817	0.175	0.608	Abs
EUAS3	30	Power	2.636	0.00	3918.488	9	3.138	0.092	0.816	Abs
EUAS3	30	Power	2.841	0.00	0.016	10	2.812	0.111	0.515	Prop
EUAS3	35	LogNorm	6.706	1.82	90.625	15	-2.990	0.163	0.711	Abs
EUAS3	40	Exp	0.002	0.00	1077.212	12	-0.314	0.731	0.513	Abs
EUAS3	40	Exp	275.466	0.00	0.012	7	-0.202	0.559	0.516	Prop

Region	threshold	model_name	par1	par2	X_{min}	n	LRT	p-value	AICc_weight	group
NA1	20	Power	2.202	0.00	535708.579	14	3.668	0.149	0.780	Abs
NA1	20	Power	2.222	0.00	0.014	14	3.894	0.150	0.785	Prop
NA1	25	Power	2.205	0.00	717227.730	8	1.196	0.479	0.682	Abs
NA1	25	Power	2.266	0.00	0.022	8	1.593	0.364	0.735	Prop
NA1	30	Power	2.499	0.00	1012371.676	11	0.256	0.860	0.493	Abs
NA1	30	Power	2.573	0.00	0.035	11	0.677	0.642	0.575	Prop
NA1	35	Power	6.793	0.00	4777863.363	6	0.011	0.973	0.483	Abs
NA1	35	Power	6.989	0.00	0.182	6	0.135	0.672	0.475	Prop
NA1	40	Power	2.659	0.00	947071.521	8	0.889	0.471	0.638	Abs
NA1	40	Power	2.472	0.00	0.036	9	0.391	0.757	0.531	Prop
NA5	20	Power	3.839	0.00	27343.399	8	0.067	0.917	0.477	Abs
NA5	20	Power	4.286	0.00	0.067	8	0.578	0.427	0.583	Prop
NA5	25	Power	6.558	0.00	43897.199	6	0.009	0.976	0.482	Abs
NA5	30	Exp	0.000	0.00	41295.732	8	-0.261	0.652	0.523	Abs
NA5	30	Power	3.598	0.00	0.115	8	0.021	0.979	0.467	Prop
NA5	35	Exp	8.540	0.00	0.080	10	-1.663	0.156	0.738	Prop
NA5	40	Exp	0.000	0.00	40689.945	7	-0.243	0.597	0.526	Abs
NA5	40	Exp	10.281	0.00	0.111	11	-1.351	0.197	0.691	Prop
OC1	20	Power	3.456	0.00	0.015	6	2.293	0.004	0.844	Prop
OC1	25	Exp	0.000	0.00	18629.161	10	-0.767	0.267	0.599	Abs
OC1	25	Exp	297.422	0.00	0.009	10	-0.450	0.441	0.547	Prop
OC1	30	Exp	0.000	0.00	18183.975	8	-0.220	0.740	0.513	Abs
OC1	30	Exp	200.755	0.00	0.008	11	-0.047	0.959	0.458	Prop
OC1	35	Exp	0.000	0.00	5980.411	9	-0.195	0.891	0.497	Abs
OC1	35	Exp	175.829	0.00	0.004	9	-0.201	0.884	0.497	Prop
OC1	40	Exp	190.930	0.00	0.006	10	-1.516	0.104	0.696	Prop
OC2	20	Exp	0.000	0.00	25376.671	12	-2.225	0.157	0.792	Abs
OC2	20	Exp	74.562	0.00	0.007	12	-2.190	0.167	0.789	Prop
OC2	25	Exp	0.000	0.00	81336.170	7	-0.352	0.476	0.549	Abs
OC2	25	Power	5.180	0.00	0.024	6	0.013	0.979	0.483	Prop
OC2	30	Exp	58.283	0.00	0.009	12	-2.280	0.170	0.808	Prop
OC2	35	Exp	117.828	0.00	0.031	7	-0.077	0.876	0.489	Prop
OC2	40	Exp	58.605	0.00	0.024	7	-0.618	0.462	0.608	Prop
OC3	20	Power	4.131	0.00	70028.204	10	0.626	0.416	0.555	Abs
OC3	20	Power	3.959	0.00	0.020	10	0.662	0.396	0.582	Prop
OC3	25	Exp	0.000	0.00	112047.971	7	-0.137	0.772	0.502	Abs
OC3	25	Exp	71.820	0.00	0.032	7	-0.037	0.947	0.479	Prop
OC3	30	Exp	0.000	0.00	96645.329	8	-0.684	0.336	0.606	Abs
OC3	30	Exp	44.874	0.00	0.029	8	-0.501	0.516	0.572	Prop
OC3	35	Exp	31.133	0.00	0.012	11	-1.356	0.526	0.716	Prop
OC3	40	Exp	0.000	0.00	98492.498	10	-0.081	0.952	0.462	Abs
OC3	40	Power	2.527	0.00	0.033	10	0.268	0.845	0.499	Prop
OC4	20	Exp	0.000	0.00	45830.058	8	-0.030	0.977	0.465	Abs
OC4	20	Power	3.072	0.00	0.029	7	0.760	0.493	0.620	Prop
OC4	25	Exp	0.000	0.00	26431.523	10	-0.811	0.613	0.613	Abs
OC4	25	Exp	31.567	0.00	0.015	10	-0.715	0.657	0.591	Prop
OC4	30	Power	2.033	0.00	24594.259	10	0.432	0.807	0.517	Abs
OC4	30	Power	2.027	0.00	0.014	10	0.669	0.718	0.559	Prop
OC4	35	Exp	0.000	0.00	77027.104	7	-0.025	0.979	0.475	Abs
OC4	35	Power	3.151	0.00	0.049	7	0.228	0.812	0.520	Prop
OC4	40	Exp	26.509	0.00	0.077	5	-0.113	0.807	0.518	Prop
OC5	20	Power	2.919	0.00	11334.733	11	0.915	0.463	0.617	Abs

Region	threshold	model_name	par1	par2	X_{min}	n	LRT	p-value	AICc_weight	group
OC5	20	Power	2.889	0.00	0.014	11	0.926	0.451	0.619	Prop
OC5	25	Power	3.371	0.00	20224.157	11	0.288	0.767	0.506	Abs
OC5	25	Power	3.348	0.00	0.027	11	0.303	0.760	0.509	Prop
OC5	30	Power	8.498	0.00	49471.409	5	0.270	0.226	0.544	Abs
OC5	30	Power	7.499	0.00	0.068	5	0.354	0.148	0.574	Prop
OC5	35	Exp	0.000	0.00	14464.290	12	-1.427	0.392	0.714	Abs
OC5	35	Exp	25.260	0.00	0.021	12	-1.450	0.399	0.715	Prop
OC5	40	Power	1.949	0.00	6841.959	15	0.577	0.842	0.517	Abs
OC5	40	Power	1.931	0.00	0.010	15	0.706	0.812	0.538	Prop
OC6	20	Power	5.485	0.00	47190.471	5	0.020	0.945	0.496	Abs
OC6	20	Power	4.199	0.00	0.091	9	0.458	0.540	0.492	Prop
OC6	25	Power	5.372	0.00	27816.808	7	0.183	0.716	0.500	Abs
OC6	25	Exp	31.278	0.00	0.075	6	-0.062	0.902	0.495	Prop
OC6	30	Exp	0.000	0.00	6870.874	14	-3.145	0.029	0.793	Abs
OC6	30	Exp	65.059	0.00	0.067	6	-0.212	0.434	0.528	Prop
OC6	35	Exp	52.760	0.00	0.043	7	-0.327	0.542	0.544	Prop
OC6	40	Power	4.670	0.00	9180.710	6	0.159	0.752	0.517	Abs
OC6	40	Power	4.019	0.00	0.026	8	0.060	0.920	0.476	Prop
OC7	20	Power	2.707	0.00	36111.098	8	0.845	0.511	0.631	Abs
OC7	20	Exp	10.229	0.00	0.097	8	-0.001	0.999	0.460	Prop
OC7	25	Power	1.896	0.00	5404.068	14	4.499	0.173	0.772	Abs
OC7	25	Power	1.873	0.00	0.017	14	3.158	0.247	0.735	Prop
OC7	30	Exp	0.000	0.00	41859.857	7	-0.008	0.984	0.472	Abs
OC7	30	Exp	15.825	0.00	0.138	8	-0.844	0.086	0.595	Prop
OC7	35	Exp	11.467	0.00	0.044	9	-1.659	0.273	0.765	Prop
OC7	40	Exp	0.000	0.00	1288.907	15	-1.724	0.666	0.530	Abs
OC7	40	Power	4.147	0.00	0.082	6	1.256	0.060	0.712	Prop
OC8	20	Exp	0.000	0.00	48319.705	11	-0.494	0.538	0.555	Abs
OC8	20	Power	4.231	0.00	0.115	9	0.426	0.595	0.536	Prop
OC8	25	Exp	10.816	0.00	0.075	10	-0.756	0.575	0.615	Prop
OC8	30	Power	7.628	0.00	72921.357	5	0.215	0.462	0.530	Abs
OC8	30	Exp	35.668	0.00	0.167	7	-0.086	0.751	0.491	Prop
OC8	35	Exp	0.000	0.00	45277.477	5	-0.174	0.747	0.533	Abs
OC8	40	Power	3.668	0.00	28428.168	6	1.271	0.013	0.734	Abs
OC8	40	Power	4.178	0.00	0.096	6	0.729	0.175	0.639	Prop
SAST1	20	Exp	0.000	0.00	355101.013	13	-0.895	0.697	0.592	Abs
SAST1	20	Exp	39.269	0.00	0.007	13	-0.877	0.704	0.587	Prop
SAST1	25	Exp	0.000	0.00	220416.061	12	-1.858	0.493	0.751	Abs
SAST1	25	Exp	39.766	0.00	0.004	12	-1.947	0.471	0.761	Prop
SAST1	30	Power	2.486	0.00	503871.186	9	3.586	0.070	0.701	Abs
SAST1	30	Power	2.479	0.00	0.011	9	3.712	0.065	0.726	Prop
SAST1	35	Power	3.061	0.00	2702451.796	13	2.278	0.053	0.744	Abs
SAST1	35	Power	3.042	0.00	0.064	13	2.430	0.038	0.501	Prop
SAST1	40	Power	7.009	0.00	6241174.806	8	0.189	0.585	0.462	Abs
SAST1	40	Power	6.734	0.00	0.156	8	0.264	0.479	0.514	Prop
SAST2	20	Power	3.563	0.00	78547.171	8	0.612	0.433	0.465	Abs
SAST2	20	Power	3.527	0.00	0.215	9	0.270	0.779	0.514	Prop
SAST2	25	Power	1.943	0.00	10044.479	14	2.618	0.354	0.729	Abs
SAST2	25	Power	1.846	0.00	0.030	14	0.427	0.880	0.476	Prop
SAST2	30	Power	6.000	0.00	22320.877	7	0.783	0.133	0.566	Abs
SAST2	35	Exp	0.000	0.00	9319.879	11	-0.350	0.708	0.523	Abs
SAST2	35	Exp	36.279	0.00	0.052	10	-0.930	0.154	0.617	Prop

Region	threshold	model_name	par1	par2	X_{min}	n	LRT	p-value	AICc_weight	group
SAST2	40	Exp	0.001	0.00	3376.195	8	-0.299	0.626	0.531	Abs
SAT1	20	Exp	0.000	0.00	63276.634	11	-0.243	0.782	0.503	Abs
SAT1	20	Power	3.448	0.00	0.053	11	0.396	0.704	0.528	Prop
SAT1	25	Exp	0.000	0.00	21411.259	11	-1.387	0.442	0.714	Abs
SAT1	25	Exp	20.504	0.00	0.019	11	-1.286	0.486	0.696	Prop
SAT1	30	Power	3.217	0.00	32787.253	10	1.244	0.230	0.662	Abs
SAT1	30	Power	3.129	0.00	0.033	10	1.924	0.087	0.742	Prop
SAT1	35	Power	3.539	0.00	47597.382	9	0.410	0.563	0.542	Abs
SAT1	35	Power	3.210	0.00	0.050	9	0.647	0.446	0.587	Prop
SAT1	40	Exp	0.000	0.00	60255.509	7	-0.418	0.367	0.561	Abs
SAT1	40	Power	4.661	0.00	0.080	6	0.124	0.813	0.509	Prop
SEAS1	20	Exp	0.000	0.00	400966.834	11	-1.225	0.437	0.689	Abs
SEAS1	20	Exp	31.333	0.00	0.020	11	-1.280	0.419	0.702	Prop
SEAS1	25	Power	1.832	0.00	314228.089	13	0.246	0.915	0.436	Abs
SEAS1	25	Power	2.024	0.00	0.024	12	1.237	0.583	0.638	Prop
SEAS1	30	Power	2.326	0.00	325455.208	11	3.298	0.172	0.797	Abs
SEAS1	30	Power	2.677	0.00	0.034	7	2.454	0.079	0.828	Prop
SEAS1	35	Exp	0.000	0.00	841123.593	5	-0.132	0.570	0.523	Abs
SEAS1	35	Exp	61.918	0.00	0.052	6	-0.357	0.391	0.559	Prop
SEAS1	40	Exp	0.000	0.00	1065197.539	7	-0.335	0.269	0.533	Abs
SEAS1	40	Exp	75.501	0.00	0.093	7	-0.109	0.645	0.495	Prop
SEAS2	20	Power	3.585	0.00	34327.805	7	1.125	0.221	0.691	Abs
SEAS2	20	Power	3.390	0.00	0.082	7	1.276	0.210	0.676	Prop
SEAS2	25	Power	6.521	0.00	66017.561	6	0.977	0.008	0.673	Abs
SEAS2	25	Power	5.658	0.00	0.171	7	1.017	0.020	0.613	Prop
SEAS2	30	Exp	45.179	0.00	0.271	8	-0.029	0.841	0.471	Prop
SEAS2	40	Power	2.874	0.00	15568.943	12	1.723	0.149	0.709	Abs
SEAS2	40	Power	2.832	0.00	0.057	12	1.087	0.359	0.637	Prop

Table S5: Quantil regressions of the fluctuations of the largest patch vs year, for 10% and 90% quantils at different pixel thresholds. Only the significant quantils are showed. The column group signals if the fluctuations are relative to total forest area RS_{max} (prop) or absolute S_{max} (Abs).

Region	Threshold	Slope	StdError	t_value	p_value	tau	group
AF1	30	-0.0134	0.0053	-2.5162	0.0247	0.1	Prop
AF1	30	-0.0061	0.0025	-2.3991	0.0309	0.9	Prop
AF1	30	-243262	97427	-2.4968	0.0256	0.1	Abs
AF1	30	-121965	48406	-2.5196	0.0245	0.9	Abs
AF2	20	0.0038	0.0014	2.6847	0.0178	0.1	Prop
AF2	20	2827	1078	2.6222	0.0201	0.1	Abs
EUAS1	20	0.0072	0.0024	3.005	0.0095	0.1	Prop
EUAS1	20	388824	137508	2.828	0.0134	0.1	Abs
EUAS1	30	0.0023	0.001	2.372	0.0325	0.9	Prop
EUAS1	30	104954	43636	2.405	0.0306	0.9	Abs
EUAS1	40	-0.0073	0.003	-2.461	0.0275	0.1	Prop
EUAS1	40	-223815	88877	-2.518	0.0246	0.1	Abs
EUAS2	35	-0.0046	0.0017	-2.7541	0.0155	0.1	Prop
EUAS2	35	-3770	1369	-2.7541	0.0155	0.1	Abs
EUAS2	40	-0.0054	0.0019	-2.8186	0.0137	0.1	Prop
EUAS2	40	-4196	1665	-2.5199	0.0245	0.1	Abs
NA1	20	0.0204	0.0080	2.5660	0.0224	0.1	Prop
NA1	20	0.0093	0.0023	4.1006	0.0011	0.9	Prop
NA1	20	753247	285394	2.6393	0.0194	0.1	Abs
NA1	20	375157	90502	4.1453	0.0010	0.9	Abs
NA1	25	0.0192	0.0082	2.3406	0.0346	0.1	Prop
NA1	25	0.0067	0.0018	3.7572	0.0021	0.9	Prop
NA1	25	621998	264812	2.3488	0.0340	0.1	Abs
NA1	25	240816	60083	4.0081	0.0013	0.9	Abs
NA1	30	0.0200	0.0071	2.8167	0.0137	0.1	Prop
NA1	30	568396	191424	2.9693	0.0102	0.1	Abs
OC1	25	4099	1758	2.3321	0.0351	0.9	Abs
OC1	30	0.0021	0.0006	3.4269	0.0041	0.9	Prop
OC1	30	3994	1132	3.5285	0.0033	0.9	Abs
OC1	35	0.0012	0.0005	2.5132	0.0248	0.9	Prop
OC1	35	1836	747	2.4582	0.0276	0.9	Abs
OC2	25	-0.0029	0.0011	-2.7063	0.0170	0.9	Prop
OC2	25	-11599	4540	-2.5548	0.0229	0.9	Abs
OC2	30	-0.0039	0.0013	-2.8856	0.0120	0.9	Prop
OC2	30	-14910	5158	-2.8909	0.0119	0.9	Abs
OC2	35	-0.0042	0.0015	-2.8100	0.0139	0.9	Prop
OC2	35	-15676	6597	-2.3762	0.0323	0.9	Abs
OC3	35	0.0054	0.0025	2.1548	0.0491	0.9	Prop
OC3	40	-0.0144	0.0066	-2.1859	0.0463	0.1	Prop
OC3	40	-38943	17487	-2.2270	0.0429	0.1	Abs
OC4	20	0.0074	0.0020	3.6722	0.0025	0.9	Prop
OC4	20	14008	4103	3.4142	0.0042	0.9	Abs
OC4	25	0.0075	0.0028	2.7121	0.0169	0.9	Prop
OC4	25	13656	5309	2.5724	0.0221	0.9	Abs
OC4	30	0.0104	0.0038	2.6975	0.0173	0.9	Prop
OC4	30	19189	7070	2.7140	0.0168	0.9	Abs
OC5	35	-0.0105	0.0048	-2.1867	0.0462	0.1	Prop
OC6	40	0.0087	0.0031	2.8059	0.0140	0.9	Prop

Region	Threshold	Slope	StdError	t_value	p_value	tau	group
OC6	40	2621	941	2.7861	0.0146	0.9	Abs
SAST1	25	-0.0036	0.0015	-2.3616	0.0332	0.1	Prop
SAST1	25	-169932	77123	-2.2034	0.0448	0.1	Abs
SAST1	30	-0.0104	0.0047	-2.2017	0.0450	0.1	Prop
SAST1	30	-450641	208359	-2.1628	0.0483	0.1	Abs
SAST1	40	-0.0151	0.0063	-2.4164	0.0299	0.1	Prop
SAST2	20	0.0500	0.0226	2.2157	0.0438	0.1	Prop
SAST2	20	0.0479	0.0147	3.2548	0.0058	0.9	Prop
SAST2	20	20767	6695	3.1018	0.0078	0.9	Abs
SAST2	25	0.0419	0.0145	2.8801	0.0121	0.9	Prop
SAST2	25	15131	4830	3.1325	0.0073	0.9	Abs
SAST2	30	4722	1786	2.6438	0.0193	0.9	Abs
SAST2	40	0.0044	0.0018	2.4791	0.0265	0.9	Prop
SAST2	40	932	379	2.4574	0.0276	0.9	Abs
SAT1	20	0.0124	0.0055	2.2481	0.0412	0.1	Prop
SAT1	25	0.0098	0.0032	3.1109	0.0077	0.1	Prop
SAT1	25	8772	2529	3.4687	0.0038	0.1	Abs
SAT1	30	0.0125	0.0028	4.4724	0.0005	0.1	Prop
SAT1	30	11009	2644	4.1636	0.0010	0.1	Abs
SAT1	35	0.0124	0.0043	2.8571	0.0127	0.1	Prop
SAT1	35	9171	3468	2.6444	0.0192	0.1	Abs
SEAS1	20	0.0072	0.0032	2.2470	0.0413	0.1	Prop
SEAS1	25	0.0112	0.0033	3.3941	0.0044	0.9	Prop
SEAS1	25	234148	68433	3.4216	0.0041	0.9	Abs
SEAS2	20	0.0264	0.0098	2.6911	0.0176	0.1	Prop
SEAS2	20	10358	3856	2.6861	0.0177	0.1	Abs
SEAS2	25	0.0422	0.0119	3.5537	0.0032	0.1	Prop
SEAS2	25	14635	4485	3.2634	0.0057	0.1	Abs
SEAS2	30	0.0461	0.0212	2.1696	0.0477	0.1	Prop
SEAS2	30	0.0535	0.0073	7.2819	0	0.9	Prop
SEAS2	30	16907	2520	6.7096	0	0.9	Abs
SEAS2	40	0.0303	0.0069	4.3779	0.0006	0.9	Prop
SEAS2	40	8337	2146	3.8854	0.0016	0.9	Abs

Table S6: Unbiased estimation of Skewness of fluctuations of the largest patch S_{max} and fluctuations relative to total forest area RS_{max}. The regions are: AF1, Africa Mainland, AF2, Madagascar; EUAS1 Eurasia mainland; EUAS2 Japan; EUAS3 Great Britain; NA1 North America mainland; NA5 Newfoundland; OC1 Australia mainland; OC2 New Guinea; OC3 Malaysia/Kalimantan; OC4 Sumatra; OC5 Sulawesi; OC6 New Zealand south island; OC7 Java; OC8 New Zealand north island; SAST1 South America, Tropical and subtropical forest up to Mexico; SAST2 Cuba; SAT1 South America, Temperate forest; SEAS1 Southeast Asia mainland; SEAS2 Philippines.

Region	Threshold	S_{max} Skewness	RS_{max} Skewness
AF1	20	0.1498	0.0033
AF1	25	0.1780	-0.0354
AF1	30	-1.3442	-1.4653
AF1	35	-1.1540	-1.2117
AF1	40	0.0455	0.0140
AF2	20	-0.2313	-0.4461
AF2	25	-2.0604	-2.2019
AF2	30	0.0009	-0.1049
AF2	35	-0.0126	-0.1088
AF2	40	0.8399	0.7226
EUAS1	20	-0.447	-0.5015
EUAS1	25	0.8475	0.8054
EUAS1	30	0.4021	0.3141
EUAS1	35	0.4748	0.3943
EUAS1	40	-1.242	-1.316
EUAS2	20	0.1946	0.1140
EUAS2	25	0.3519	0.2777
EUAS2	30	0.0371	-0.0520
EUAS2	35	-0.2567	-0.3913
EUAS2	40	-0.3727	-0.5030
EUAS3	20	1.4159	1.0833
EUAS3	25	0.0574	-0.1759
EUAS3	30	3.0200	2.2396
EUAS3	35	2.9008	2.0427
EUAS3	40	0.4185	0.1860
NA1	20	-2.0879	-2.2895
NA1	25	-2.2338	-2.4465
NA1	30	-1.5664	-1.6340
NA1	35	-0.1086	-0.1448
NA1	40	2.0457	1.9814
NA5	20	-0.1270	-0.4245
NA5	25	-0.0187	-0.2326
NA5	30	-0.5825	-0.7970
NA5	35	-0.4426	-0.7516
NA5	40	0.0867	-0.1053
OC1	20	2.8348	2.7517
OC1	25	0.2592	0.1912
OC1	30	0.2425	0.0920
OC1	35	-0.7471	-0.8033
OC1	40	-0.3734	-0.4337
OC2	20	-0.1133	-0.2050
OC2	25	-0.0158	-0.1003
OC2	30	0.1862	0.1214
OC2	35	0.0714	-0.0124
OC2	40	-0.1522	-0.2847

Region	Threshold	S_{max} Skewness	RS_{max} Skewness
OC3	20	-0.6080	-0.6896
OC3	25	-0.6624	-0.7643
OC3	30	-0.8464	-0.9747
OC3	35	-0.8737	-1.0147
OC3	40	-1.3764	-1.5649
OC4	20	-1.1564	-1.3846
OC4	25	-0.2412	-0.5887
OC4	30	-0.9246	-1.4226
OC4	35	-0.5404	-0.8483
OC4	40	-0.7905	-1.0301
OC5	20	-1.4628	-1.4542
OC5	25	-1.0069	-1.0117
OC5	30	-0.1198	-0.2281
OC5	35	0.0013	-0.1880
OC5	40	0.2035	0.0323
OC6	20	-0.5538	-0.7665
OC6	25	-0.6925	-0.8074
OC6	30	-0.3390	-0.4656
OC6	35	0.5687	0.3553
OC6	40	0.4182	0.3024
OC7	20	2.0236	1.6945
OC7	25	2.6010	1.7756
OC7	30	0.1480	-0.1981
OC7	35	1.2352	0.3723
OC7	40	2.9049	2.0105
OC8	20	0.2007	-0.1078
OC8	25	0.1006	-0.3185
OC8	30	0.6030	0.3130
OC8	35	1.0935	0.6386
OC8	40	1.8703	1.3175
SAST1	20	0.2386	0.2336
SAST1	25	1.0589	1.0519
SAST1	30	-2.6255	-2.7216
SAST1	35	-1.6868	-1.7006
SAST1	40	-0.3894	-0.4192
SAST2	20	1.1417	0.5049
SAST2	25	2.2009	1.7263
SAST2	30	0.5880	0.1665
SAST2	35	1.0710	0.8241
SAST2	40	0.0373	-0.5401
SAT1	20	-0.4461	-0.6404
SAT1	25	0.4077	0.1483
SAT1	30	-1.4432	-1.6059
SAT1	35	-1.2223	-1.3809
SAT1	40	-0.5571	-0.7135
SEAS1	20	0.5641	0.2718
SEAS1	25	-1.1075	-1.3328
SEAS1	30	3.1708	3.0507
SEAS1	35	0.1358	0.0851
SEAS1	40	-0.0835	-0.1605
SEAS2	20	-1.5308	-1.6373
SEAS2	25	-0.5800	-0.6648

Region	Threshold	S_{max} Skewness	RS_{max} Skewness
SEAS2	30	0.2113	0.1517
SEAS2	35	0.8185	0.8226
SEAS2	40	1.6525	1.5996

Supplementary figures

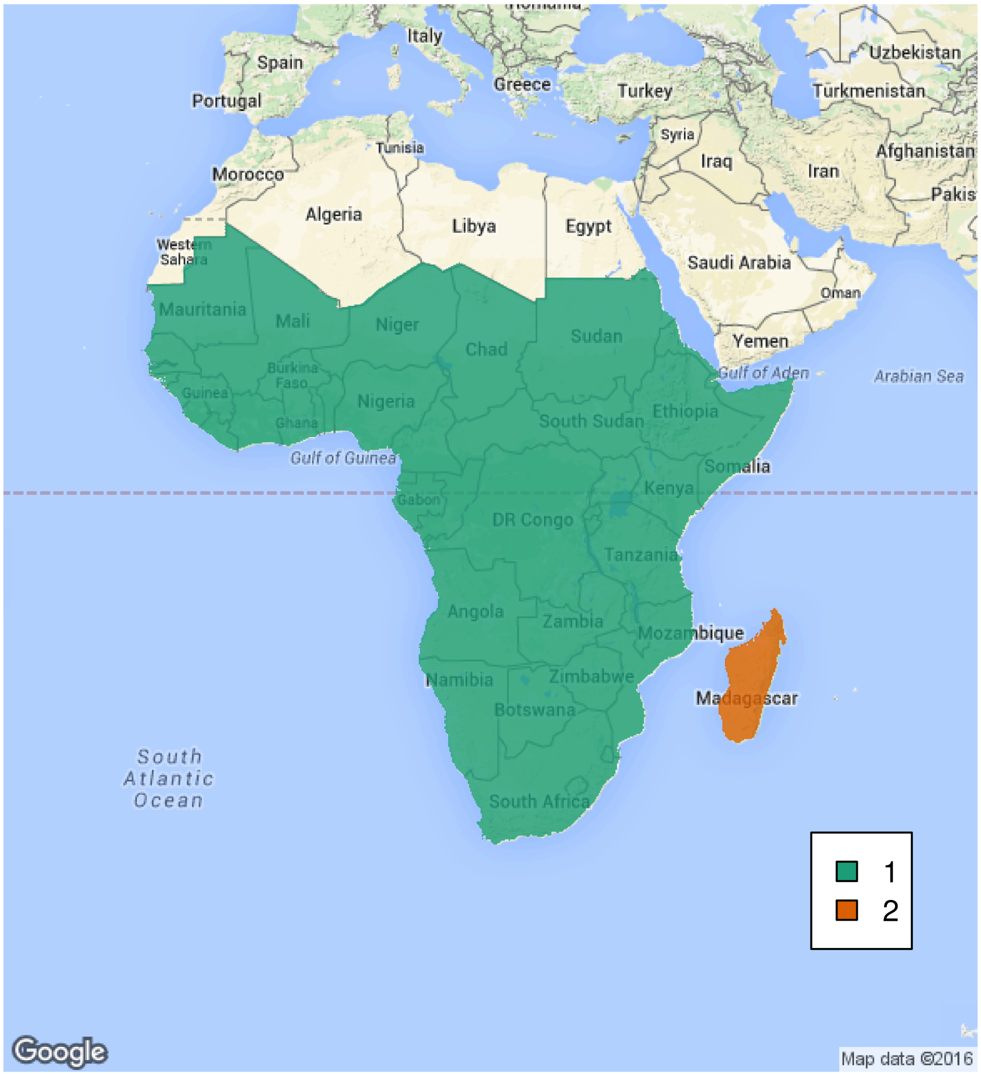

Figure S1: Regions for Africa (AF), 1 Mainland, 2 Madagascar

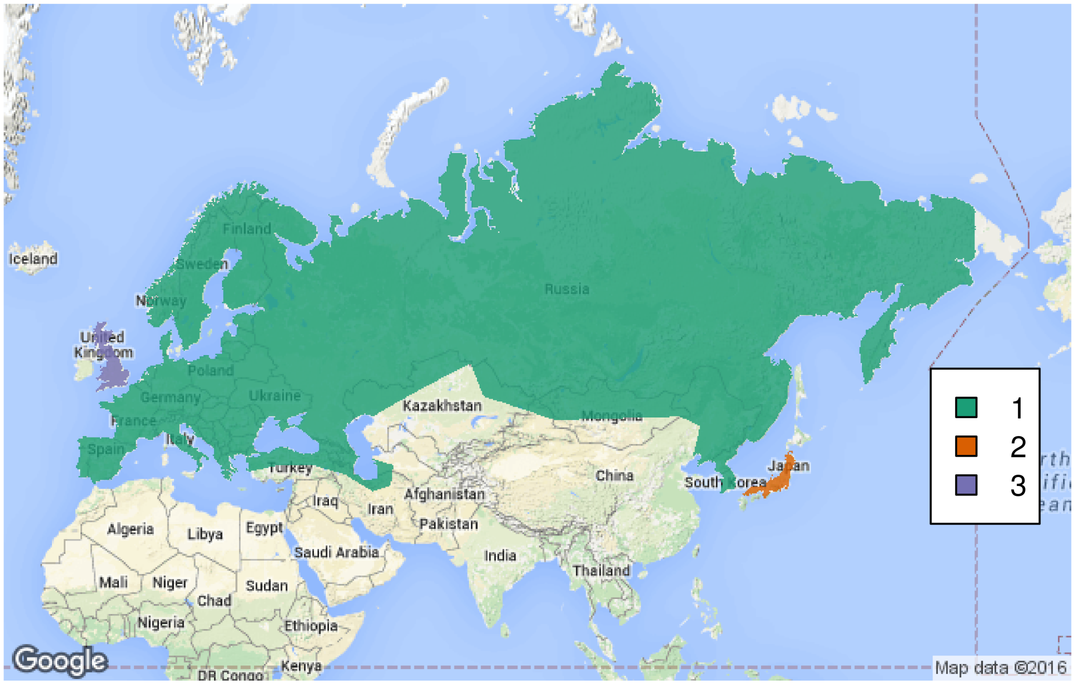

Figure S2: Regions for Eurasia (EUAS), 1 Mainland, 2 Japan, 3 Great Britain

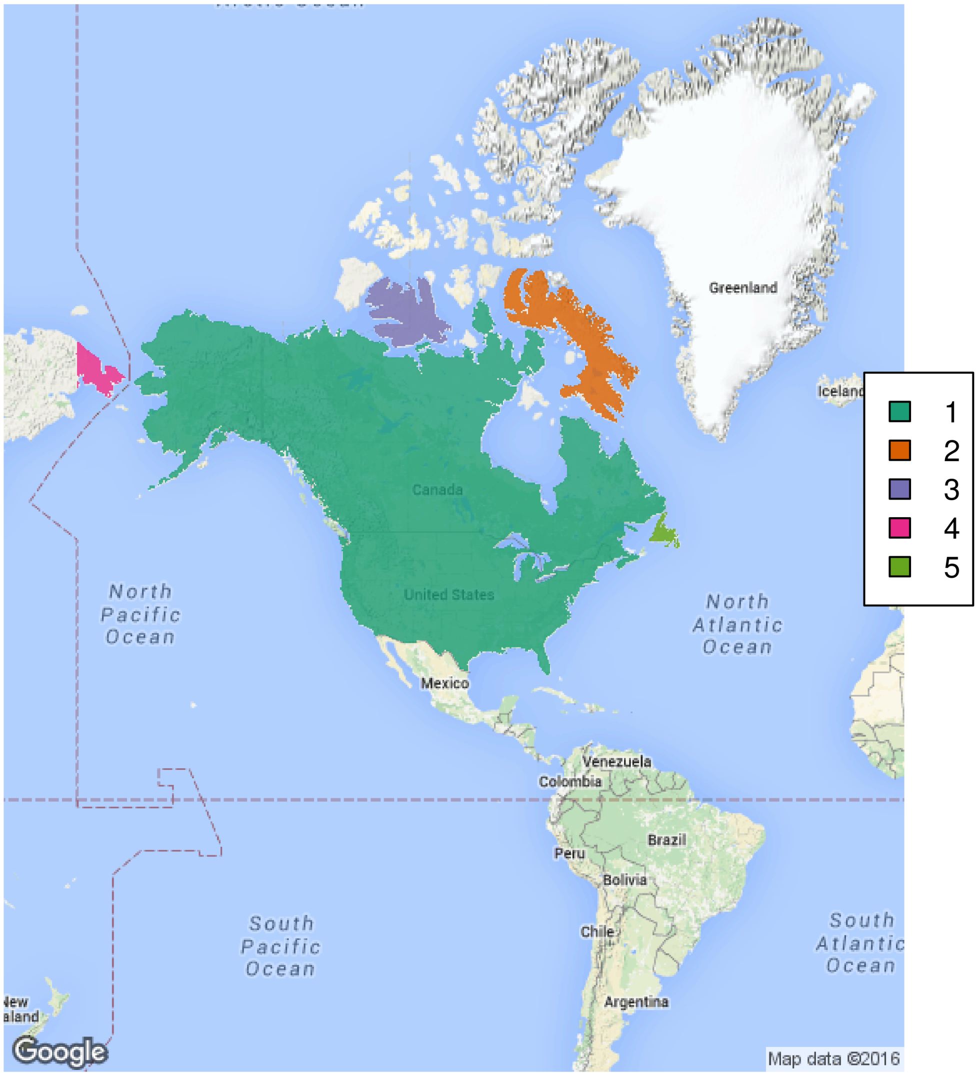

Figure S3: Regions for North America (NA), 1 Mainland, 5 Newfoundland. The other regions have a very small forest cover and were discarded for the analysis

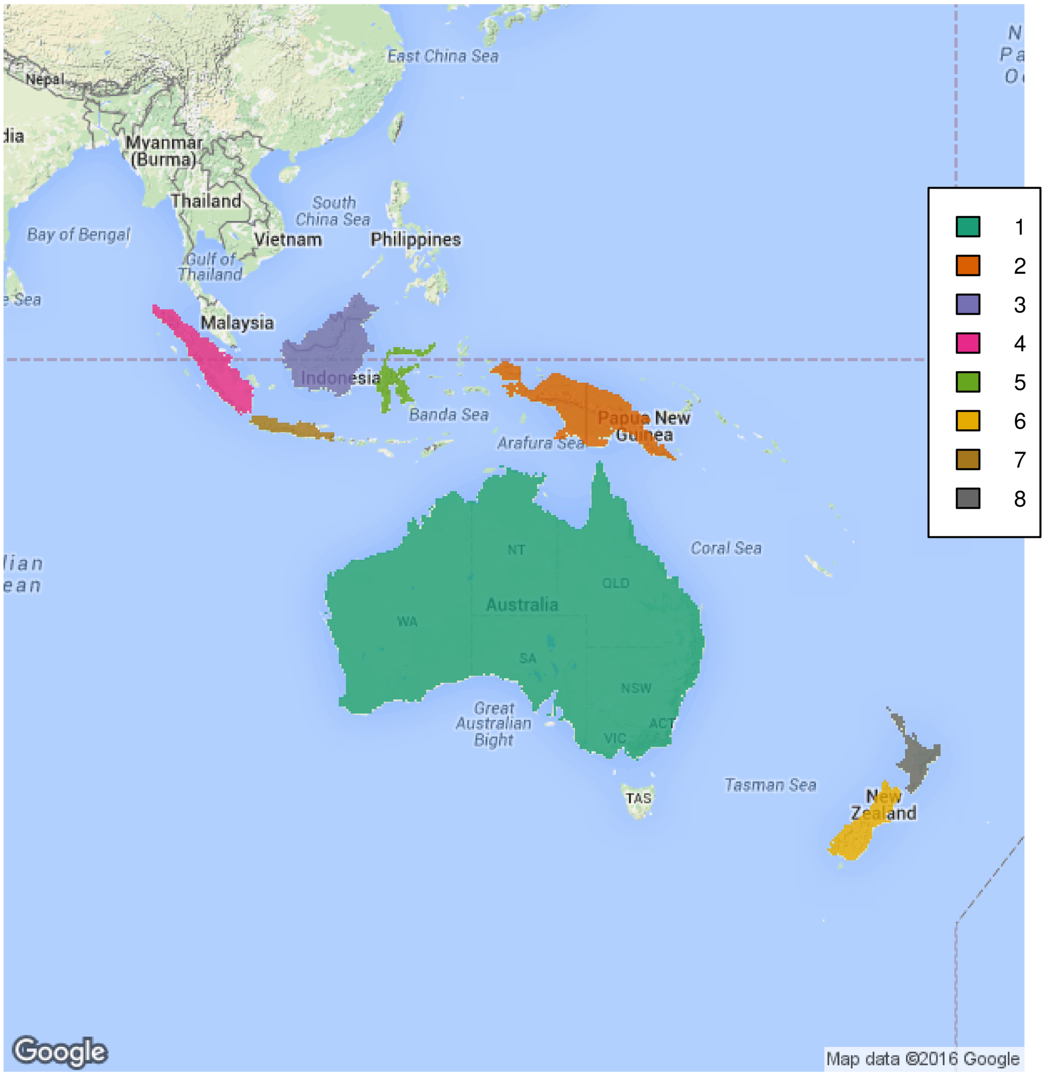

Figure S4: Regions for Australia and islands (OC), 1 Australia mainland; 2 New Guinea; 3 Malaysia/Kalimantan; 4 Sumatra; 5 Sulawesi; 6 New Zealand south island; 7 Java; 8 New Zealand north island

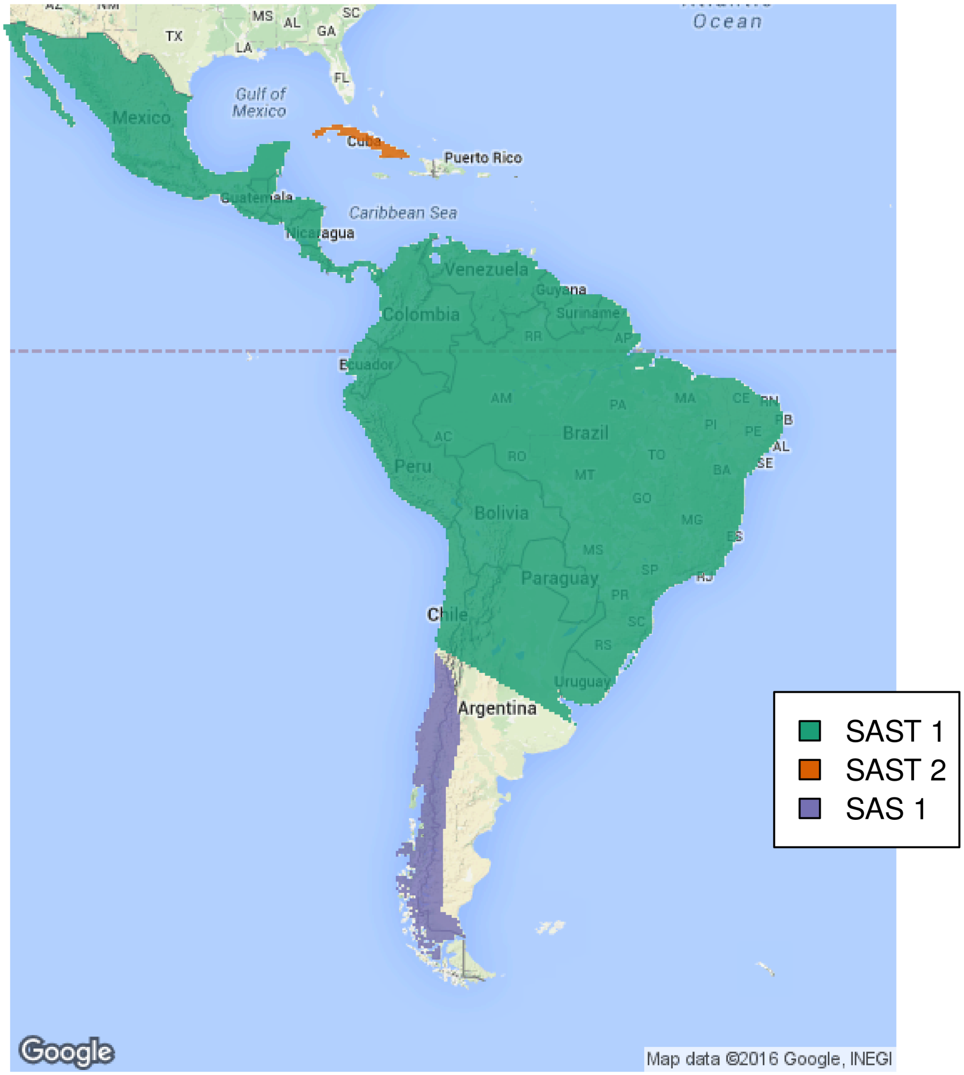

Figure S5: Regions for South America, SAST1 Tropical and subtropical forest up to Mexico; SAST2 Cuba; SAT1 South America, Temperate forest

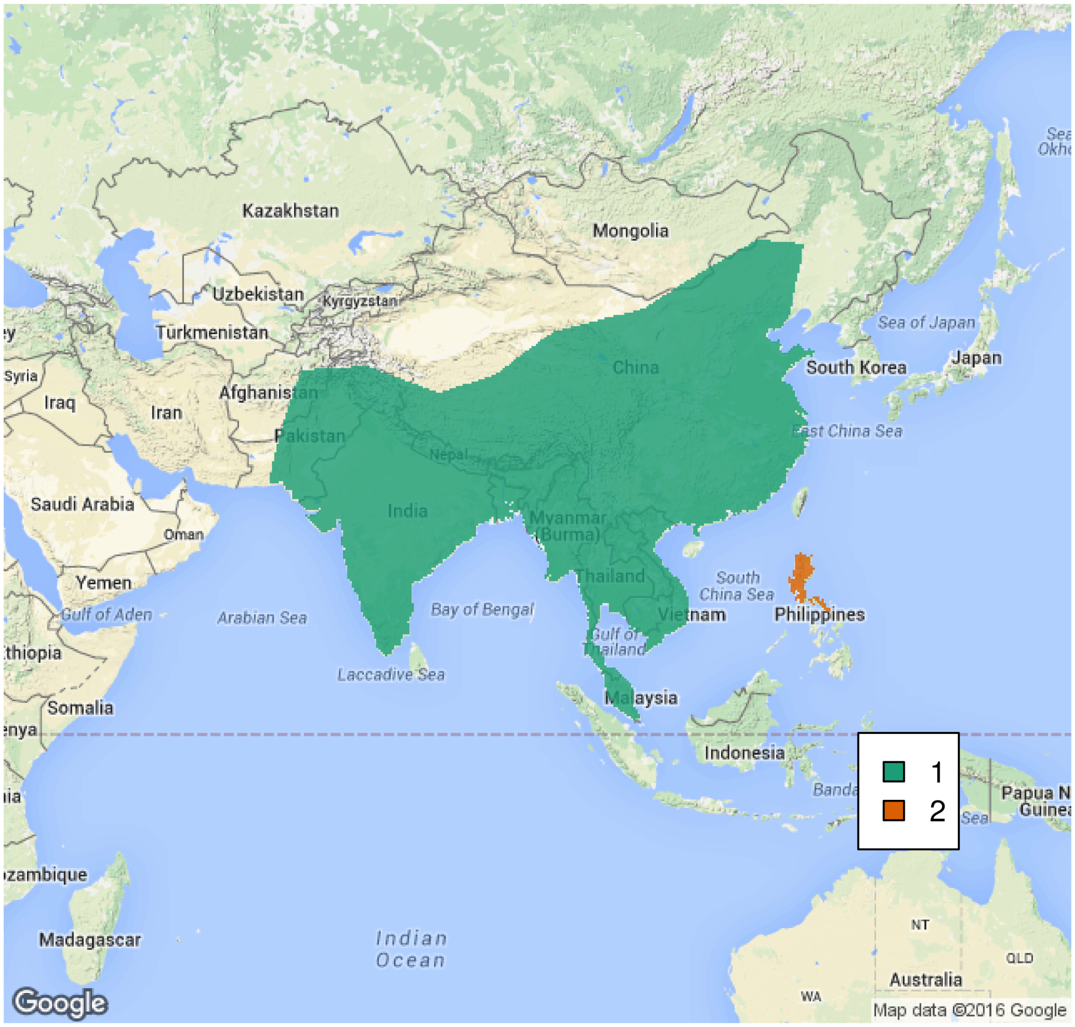

Figure S6: Regions for Southeast Asia (SEAS), 1 Mainland; 2 Philippines

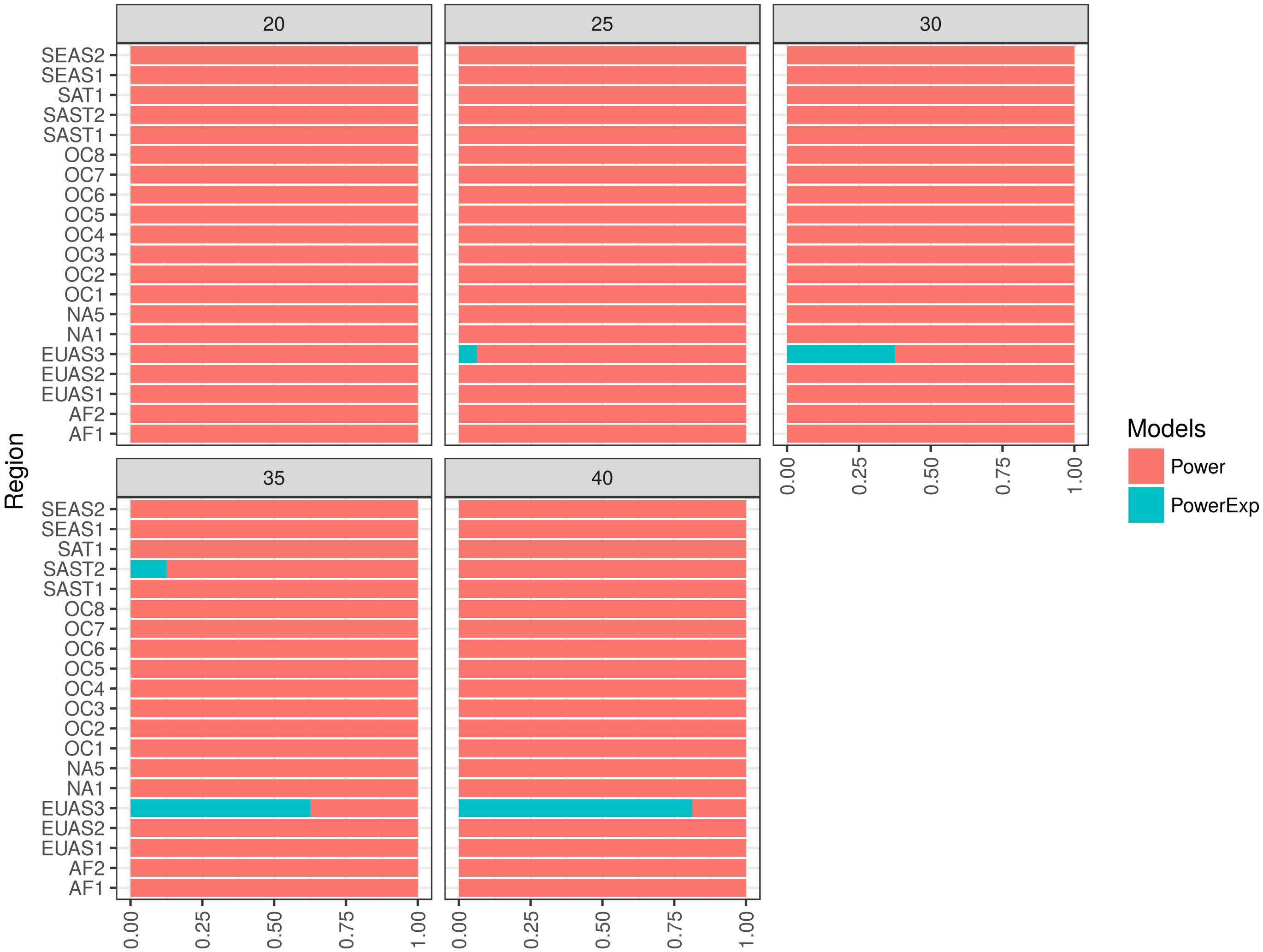

Figure S7: Proportion of best models selected for patch size distributions by region and thresholds (20,25,30,35,40); using the Akaike criterion. The regions are the same as table 1: AF1, Africa Mainland, AF2, Madagascar; EUAS1 Eurasia mainland; EUAS2 Japan; EUAS3 Great Britain; NA1 North America mainland; NA5 Newfoundland; OC1 Australia mainland; OC2 New Guinea; OC3 Malaysia/Kalimantan; OC4 Sumatra; OC5 Sulawesi; OC6 New Zealand south island; OC7 Java; OC8 New Zealand north island; SAST1 South America, Tropical and subtropical forest up to Mexico; SAST2 Cuba; SAT1 South America, Temperate forest; SEAS1 Southeast Asia mainland; SEAS2 Philippines.

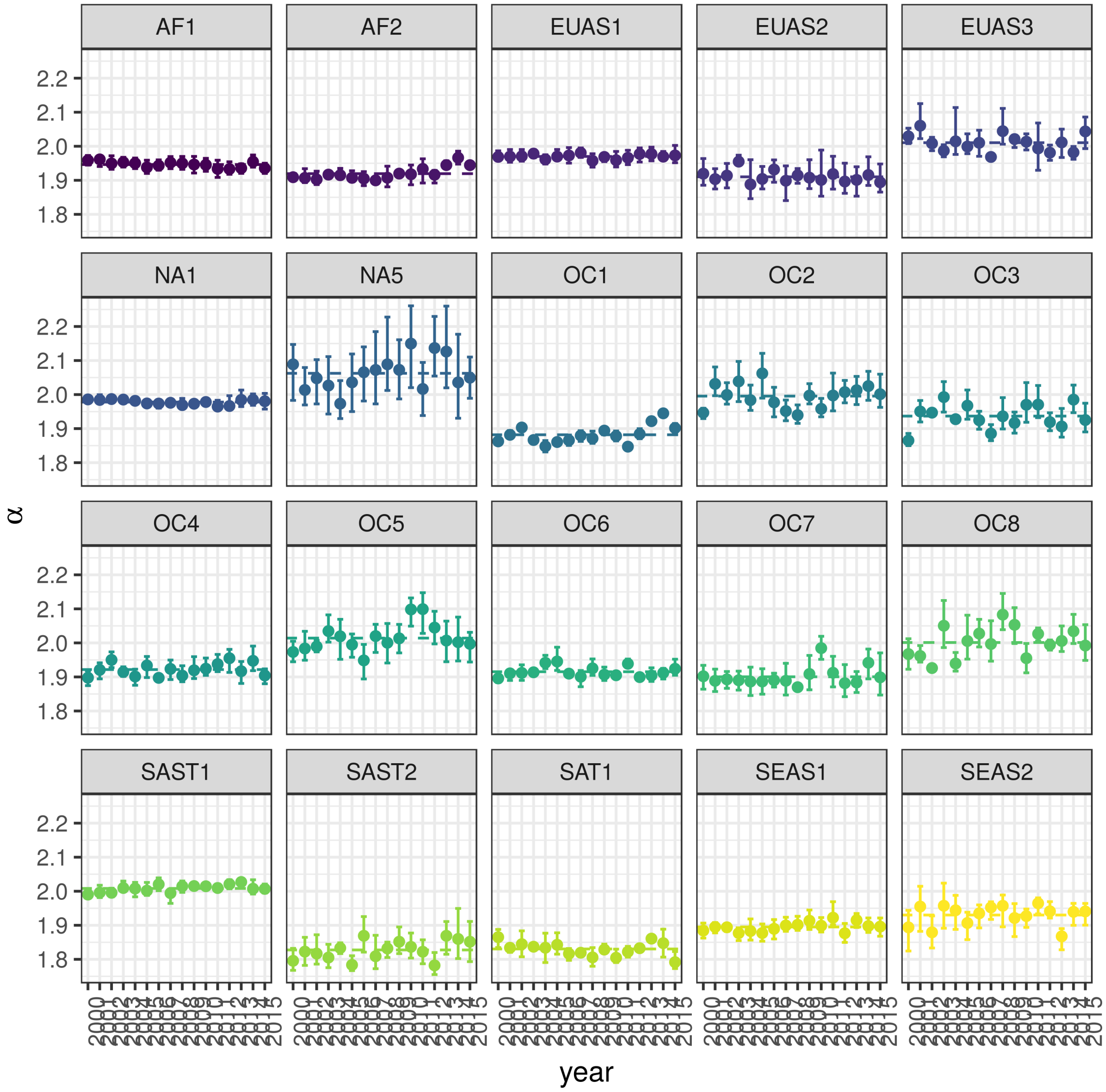

Figure S8: Power law exponents for forest patch distributions by region and year, the plot shows the fitted parameter and 95% confidence intervals estimated by bootstrap resampling across thresholds. The regions are: AF1, Africa Mainland, AF2, Madagascar; EUAS1 Eurasia mainland; EUAS2 Japan; EUAS3 Great Britain; NA1 North America mainland; NA5 Newfoundland; OC1 Australia mainland; OC2 New Guinea; OC3 Malaysia/Kalimantan; OC4 Sumatra; OC5 Sulawesi; OC6 New Zealand south island; OC7 Java; OC8 New Zealand north island; SAST1 South America, Tropical and subtropical forest up to Mexico; SAST2 Cuba; SAT1 South America, Temperate forest; SEAS1 Southeast Asia mainland; SEAS2 Philippines.

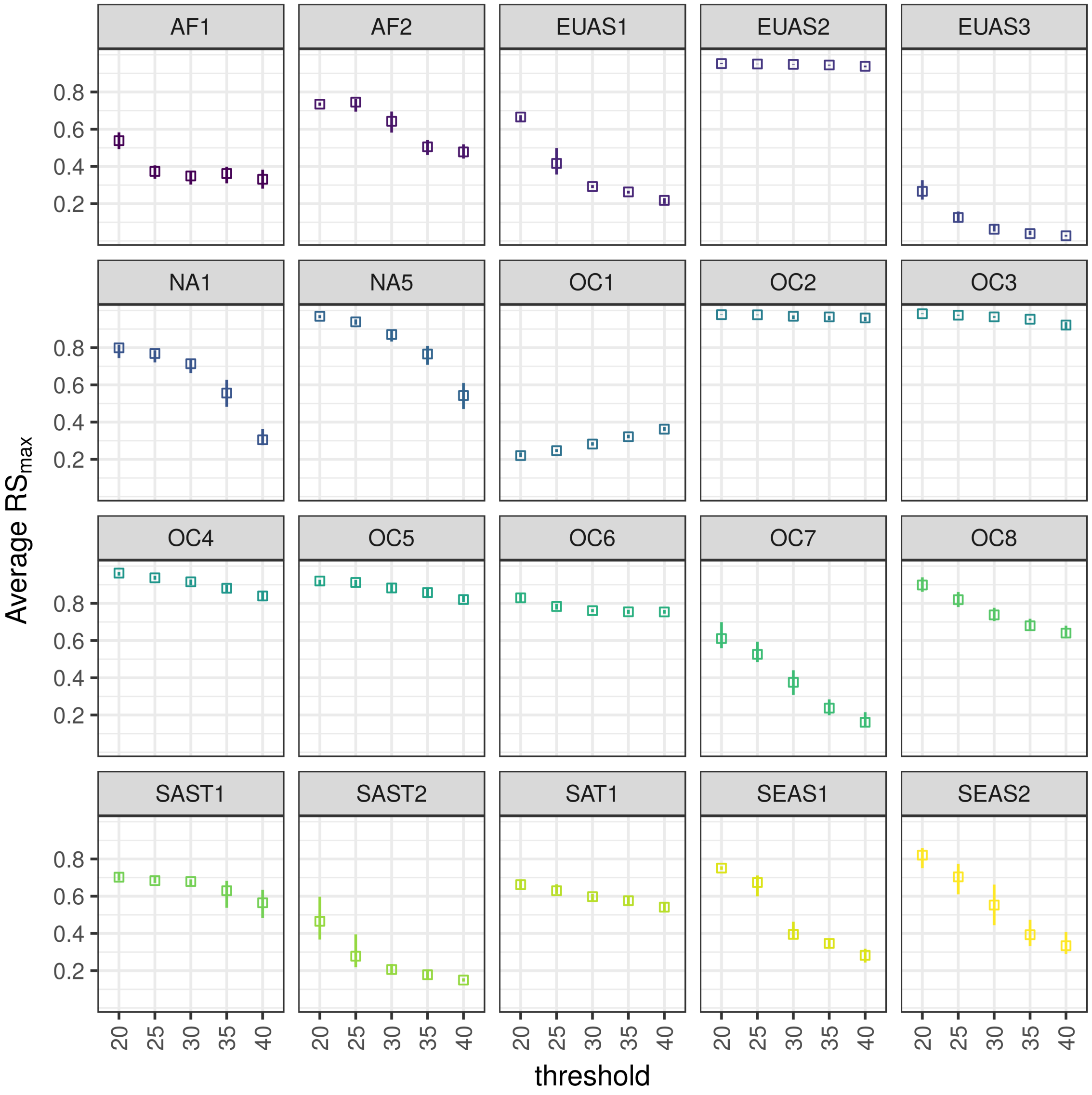

Figure S9: Average largest patch relative to total forest area RS_{max} by threshold, for all regions. The regions are: AF1, Africa Mainland, AF2, Madagascar; EUAS1 Eurasia mainland; EUAS2 Japan; EUAS3 Great Britain; NA1 North America mainland; NA5 Newfoundland; OC1 Australia mainland; OC2 New Guinea; OC3 Malaysia/Kalimantan; OC4 Sumatra; OC5 Sulawesi; OC6 New Zealand south island; OC7 Java; OC8 New Zealand north island; SAST1 South America, Tropical and subtropical forest up to Mexico; SAST2 Cuba; SAT1 South America, Temperate forest; SEAS1 Southeast Asia mainland; SEAS2 Philippines.

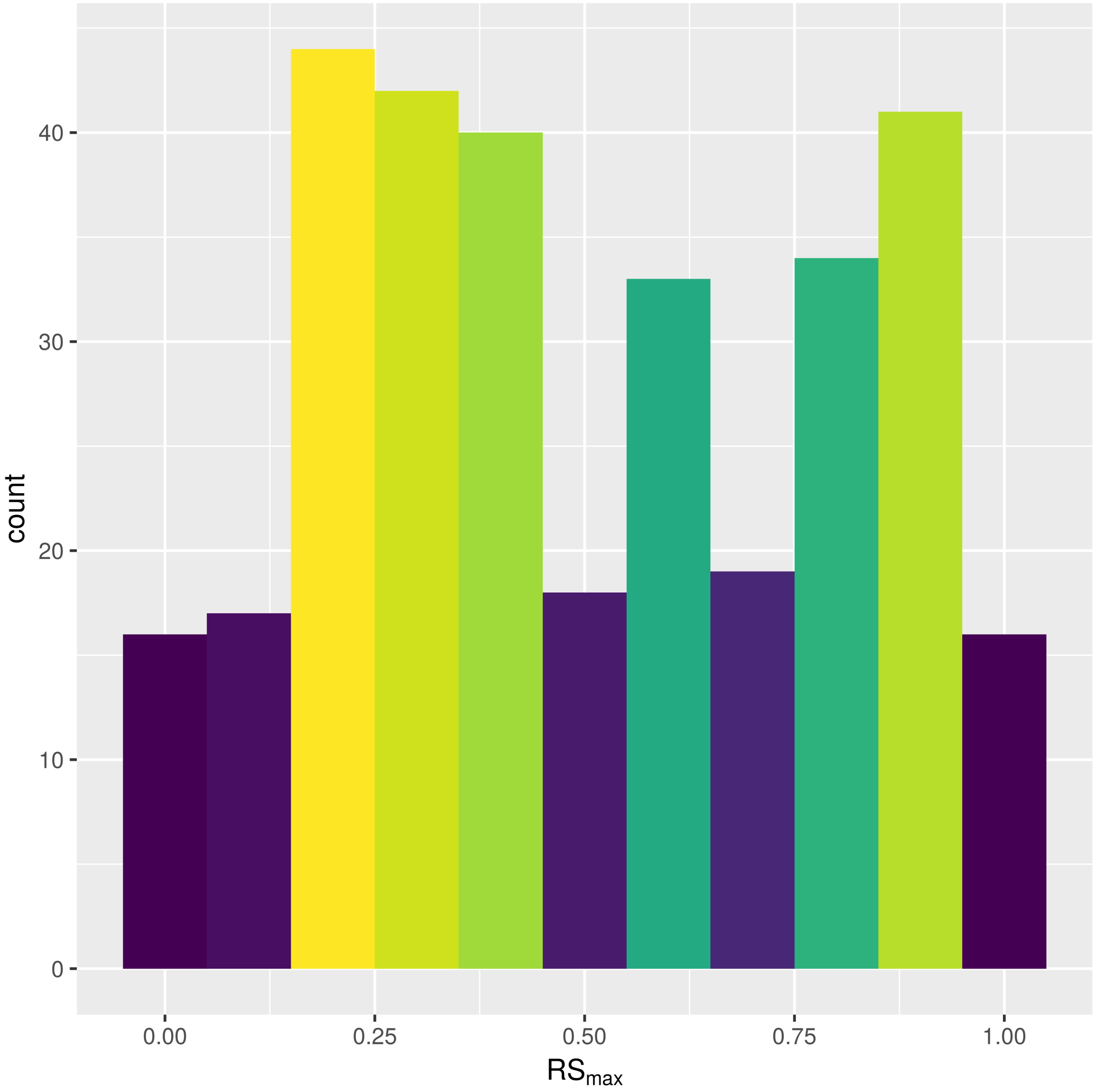

Figure S10: Frequency distribution of Largest patch proportion relative to total forest area RS_{max} calculated using a threshold of 40% of forest in each pixel to determine patches. Bimodality is observed and confirmed by the dip test (D = 0.0416, p-value = 0.0003).This indicates the existence of two states needed for a critical transition.

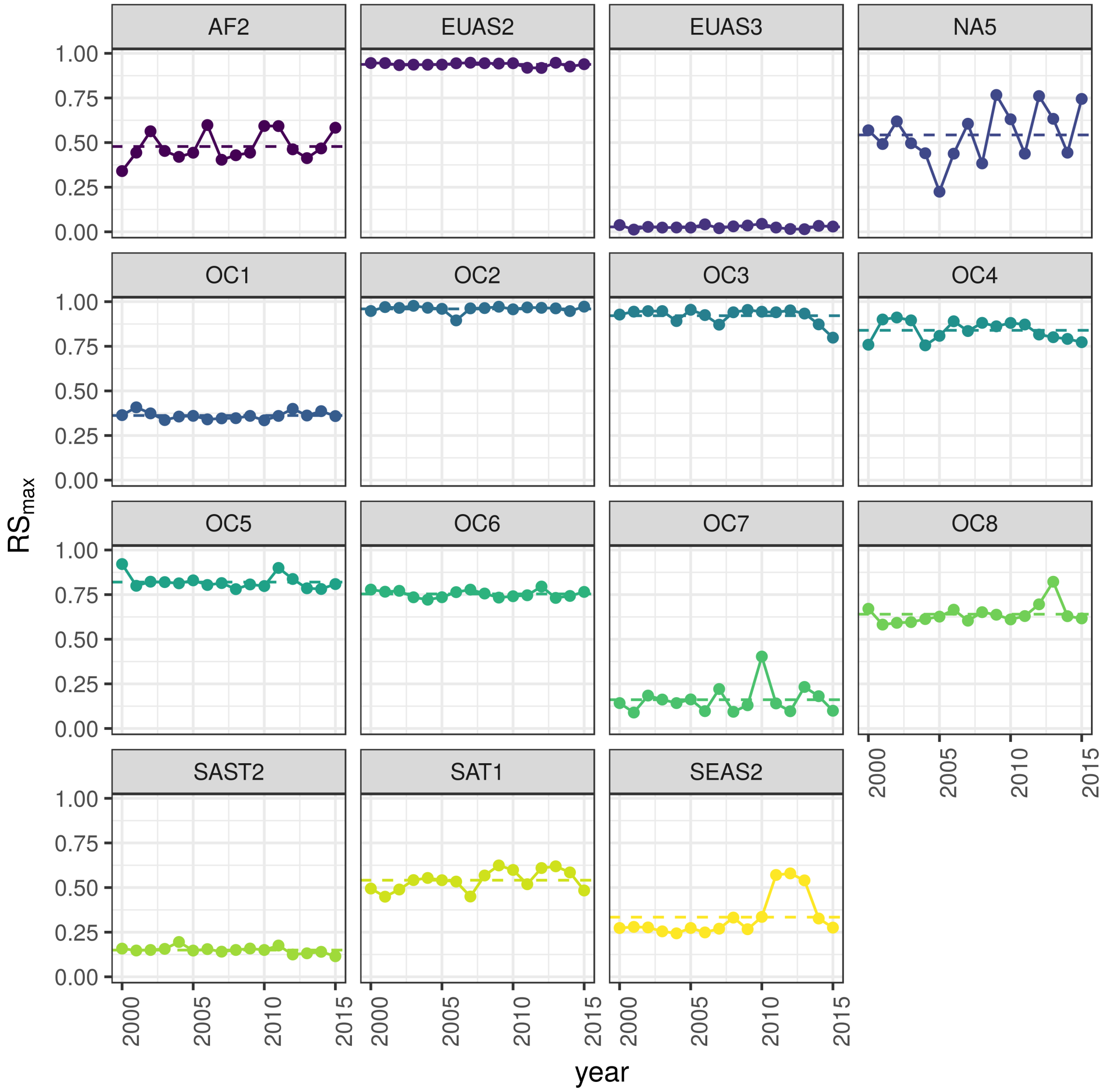

Figure S11: Largest patch relative to total forest area RS_{max} by year at 40% threshold, for regions with total forest area less than 10^7 km^2. The regions are: AF1, Africa Mainland, AF2, Madagascar; EUAS1 Eurasia mainland; EUAS2 Japan; EUAS3 Great Britain; NA1 North America mainland; NA5 Newfoundland; OC1 Australia mainland; OC2 New Guinea; OC3 Malaysia/Kalimantan; OC4 Sumatra; OC5 Sulawesi; OC6 New Zealand south island; OC7 Java; OC8 New Zealand north island; SAST1 South America, Tropical and subtropical forest up to Mexico; SAST2 Cuba; SAT1 South America, Temperate forest; SEAS1 Southeast Asia mainland; SEAS2 Philippines.

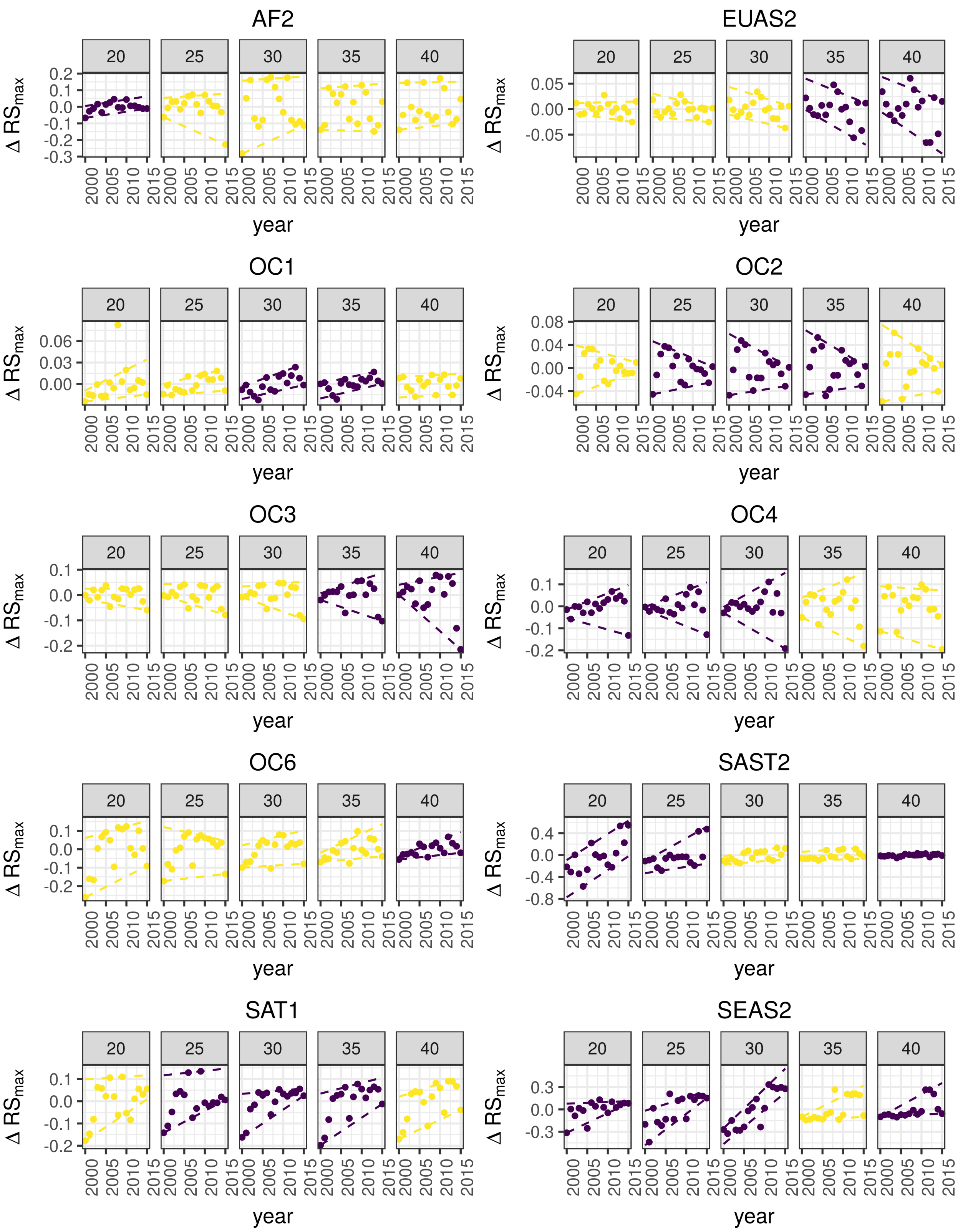

Figure S12: Fluctuations of largest patch relative to total forest area RS_{max} for regions with total forest area less than 10^7 km^2 by year and threshold. Dashed lines are quantil regressions for 90%, and 10% quantils, purple (dark) panels have significant slopes. The regions are: AF2, Madagascar; EUAS2 Japan; EUAS3 Great Britain; NA5 Newfoundland; OC1 Australia mainland; OC2 New Guinea; OC3 Malaysia/Kalimantan; OC4 Sumatra; OC5 Sulawesi; OC6 New Zealand south island; OC7 Java; OC8 New Zealand north island; Tropical and subtropical forest up to Mexico; SAST2 Cuba; SAT1 South America, Temperate forest; SEAS1 Southeast Asia mainland; SEAS2 Philippines.
